# Genomic and transcriptomic analyses of *Agrobacterium tumefaciens* S33 reveal the molecular mechanism of a novel hybrid nicotine-degrading pathway

**DOI:** 10.1038/s41598-017-05320-1

**Published:** 2017-07-06

**Authors:** Haiyan Huang, Wenjun Yu, Rongshui Wang, Huili Li, Huijun Xie, Shuning Wang

**Affiliations:** 1 State Key Laboratory of Microbial Technology, School of life science, Shandong University, Jinan, 250100 People’s Republic of China; 2 Institute of Basic Medicine, Shandong Academy of Medical Science, Jinan, 250062 People’s Republic of China; 3 Environment Research Institute, Shandong University, Jinan, 250100 People’s Republic of China

## Abstract

*Agrobacterium tumefaciens* S33 is able to degrade nicotine via a novel hybrid of the pyridine and pyrrolidine pathways. It can be utilized to remove nicotine from tobacco wastes and transform nicotine into important functionalized pyridine precursors for some valuable drugs and insecticides. However, the molecular mechanism of the hybrid pathway is still not completely clear. Here we report the genome analysis of strain S33 and its transcriptomes grown in glucose-ammonium medium and nicotine medium. The complete gene cluster involved in nicotine catabolism was found to be located on a genomic island composed of genes functionally similar but not in sequences to those of the pyridine and pyrrolidine pathways, as well as genes encoding plasmid partitioning and replication initiation proteins, conjugal transfer proteins and transposases. This suggests that the evolution of this hybrid pathway is not a simple fusion of the genes involved in the two pathways, but the result of a complicated lateral gene transfer. In addition, other genes potentially involved in the hybrid pathway could include those responsible for substrate sensing and transport, transcription regulation and electron transfer during nicotine degradation. This study provides new insights into the molecular mechanism of the novel hybrid pathway for nicotine degradation.

## Introduction

Microorganisms capable of degrading nicotine, a natural toxic alkaloid, have drawn much interest recently because they can be used to remove nicotine from tobacco industry wastes and tobacco products in an environmentally friendly fashion^1–4^. In addition, using biocatalytic processes, nicotine-degrading microorganisms transform nicotine into renewable functionalized pyridines. These pyridine precursors can be used to produce highly valuable drugs and insecticides, and often, are difficult to synthesize using traditional chemical methods^5–10^.

**Figure 1:**
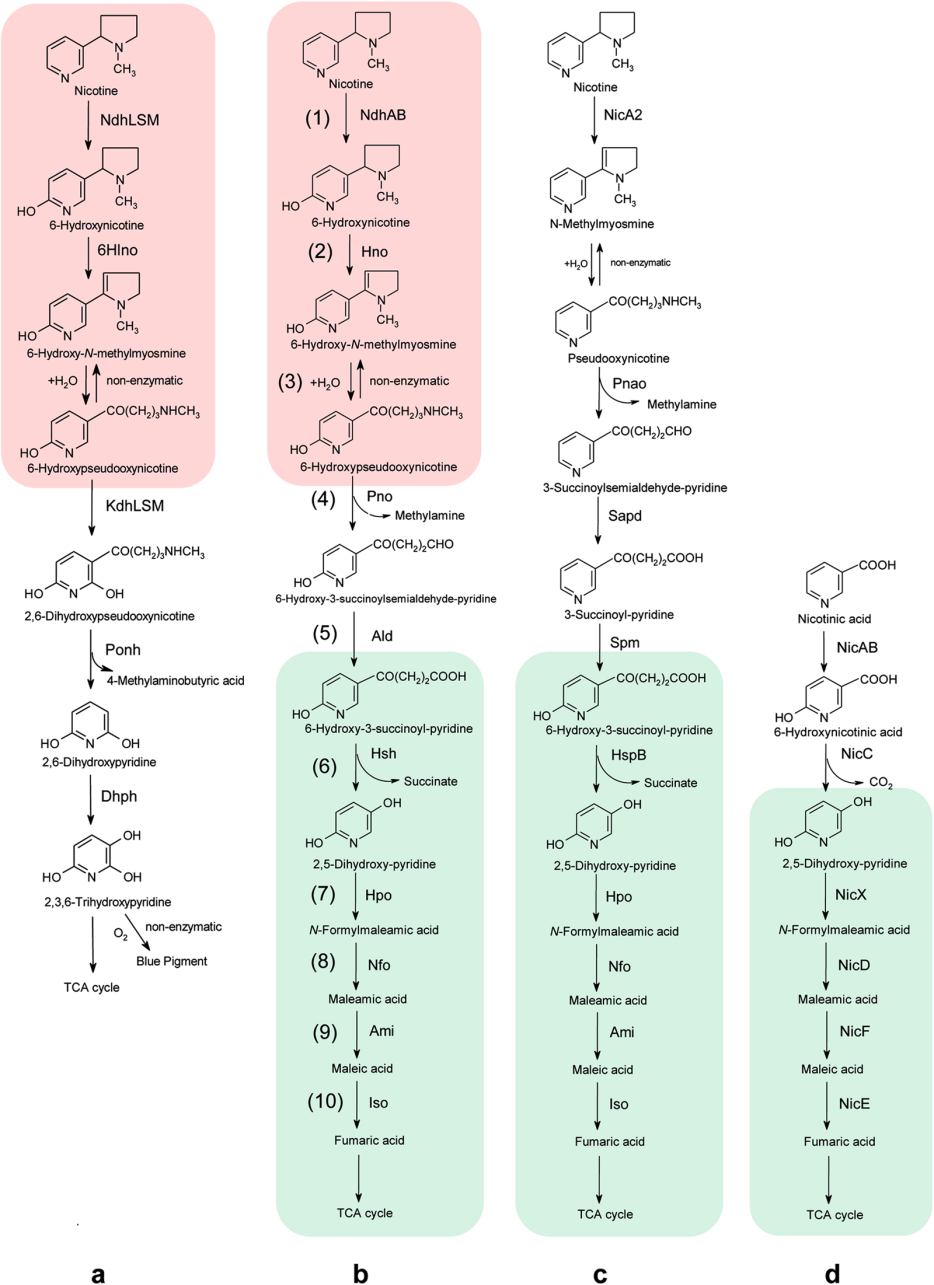
The pathways of nicotine and nicotinic acid degradation by some bacteria. (**a**) Pyridine pathway of nicotine degradation in *Arthrobacter*. Ndh, nicotine dehydrogenase; 6Hlno, 6-hydroxy-L-nicotine oxidase; Kdh, ketone dehydrogenase; Ponh, 2,6-dihydroxypseudooxynicotine hydrolase; Dhph, 2,6-dihydroxypyridine 3-hydroxylase; (**b**) A hybrid of pyridine and pyrrolidine pathways for nicotine degradation in *A*. *tumefaciens* S33 in this study. See Fig. 3 legend for enzyme abbreviations; (**c**) Pyrrolidine pathway of nicotine degradation in *Pseudomonas*. NicA, nicotine oxidoreductase; Pnao, pseudooxynicotine amidase; Sapd, 3-succinoylsemialdehyde-pyridne dehydrogenase; SpmABC, 3-succinoylpyridine monooxygenase; HspB, 6-hydroxy-3-succinoylpyridine hydroxylase; Hpo, 2,5-dihydroxypyridine dioxygenase; Nfo, *N*-formylmaleamate deformylase; Ami, maleamate amidohydrolase (amidase); Iso, maleate *cis*/*trans*-isomerase; (**d**) Nicotinic acid degradation pathway in *Pseudomonas*. NicAB, nicotinic acid hydroxylase; NicC, 6-hydroxynicotinate monooxygenase; NicX, 2,5-dihydroxypyridine dioxygenase; NicD, *N*-formylmaleamate deformylase; NicF, maleamate amidohydrolase (amidase); NicE, maleate *cis*/*trans*-isomerase. The same steps in the pyridine pathway and the hybrid pathway are shaded in red; the same steps in the hybrid pathway, the pyrrolidine pathway, and the nicotinic acid degradation pathway are shaded in blue.

A number of nicotine-degrading bacteria have been isolated from different environments, however, the biochemical pathways and molecular mechanisms involved in nicotine catabolism were well characterized only in *Arthrobacter* sp. and *Pseudomonas* sp., which degrade nicotine through the pyridine pathway and the pyrrolidine pathway (Fig. 1a and c), respectively^1, 3, 11^. Poor knowledge of nicotine catabolism in other microorganisms hinders the new technology development for disposing of tobacco wastes and utilizing tobacco and nicotine. Previously, we isolated *Agrobacterium tumefaciens* S33, which is capable of growing with nicotine as its sole carbon and nitrogen source, from the rhizospheric soil of a tobacco plant^12^. Based on the identification of chemical intermediates and enzyme assays, we discovered that *A*. *tumefaciens* S33 discomposes nicotine via a novel fused nicotine degradation pathway, formed from the standard pyridine and pyrrolidine pathways (Fig. 1b)^2^. Further, we purified and characterized the key enzymes [6-hydroxy-3-succinoylpyridine hydroxylase (Hsh), nicotine dehydrogenase (NdhAB) and 6-hydroxypseudooxynicotine oxidase (Pno)] involved in the pathway^13, 14^. Similar hybrid pathways also have been found recently in *Shinella* sp. strain HZN7^15, 16^ and *Ochrobactrum* sp. strain SJY1^17, 18^ by investigating metabolites, characterizing key enzymes [such as nicotine hydroxylase, 6-hydroxynicotine oxidase (Hno) and 2,5-dihydroxypyridine dioxygenase (Hpo)] and sequencing genomes. These studies have been integral in providing new information for a better understanding of the biochemical mechanism behind this novel hybrid nicotine degradation pathway, however, the molecular mechanism responsible for the pathway in these strains has not been completely elucidated.

In this study, we investigated the complete genome sequence of strain S33 and its transcriptomes grown in glucose-ammonium medium and nicotine medium. The gene cluster involved in nicotine catabolism was revealed to contain the genes responsible for nicotine degradation, substrate sensing and transport, transcription regulation and electron transfer. Based on genetic organization and protein sequence analysis, we postulated that this novel hybrid pathway is a result of a complicated lateral gene transfer. This study presents new results for elucidating the molecular mechanism of this novel hybrid nicotine degradation pathway.

## Results and Discussion

### Whole genome analysis

**Figure 2:**
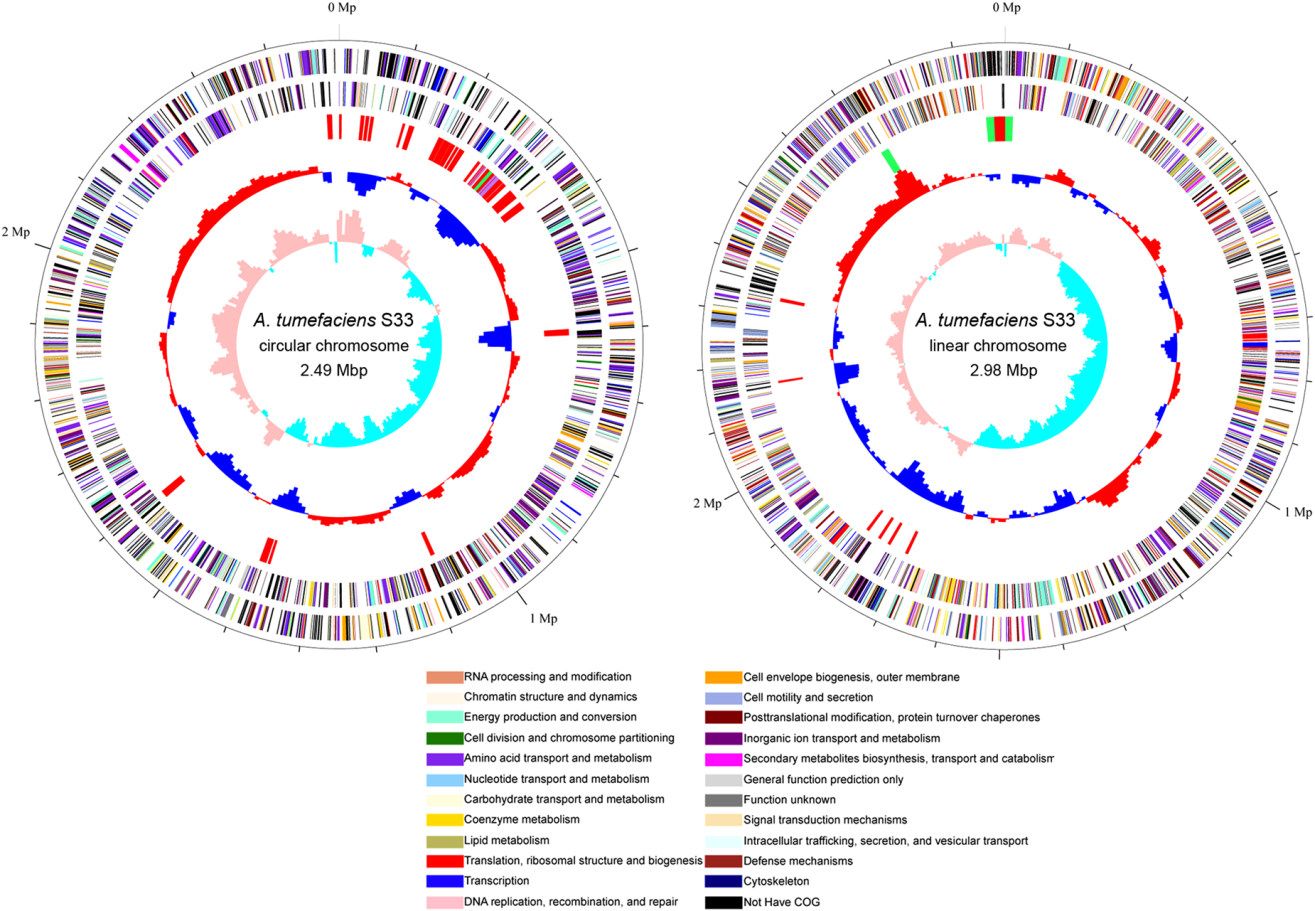
Circular representation of the circular chromosome (left) and linear chromosome (right) of *A*. *tumefaciens* S33. Outer two rings indicated genes encoded on the forward and reverse strands of the chromosome, respectively, analysed using the COG database (colours were assigned according to the colour code of the COG functional classes). The third circle indicates the predicted GIs (red), prophages (green) and the nicotine-degrading genes (purple). On the fourth ring, the deviation from the average G + C content is plotted. The most inner ring shows the value of the GC skew (G − C/G + C).

The genomic sequence of strain S33 was determined using Pacific Biosciences (PacBio) sequencing technology^19^. It is composed of 5,481,595 bases and was assembled into 2 chromosomes: one circular chromosome (2.49 Mbp; G + C content: 59.0%) and one linear chromosome (2.98 Mbp; G + C content: 59.3%) (Fig. 2). This kind of arrangement is similar to other strains of *Agrobacterium* ^20–23^; however, strain S33 does not contain any plasmids, while other *Agrobacterium* strains typically harbour at least one plasmid. Totally, there are 5,141 genes predicted in strain S33 including 4,991 protein-coding sequences (CDSs), 79 pseudogenes, 55 tRNAs and 12 rRNA genes. Using the CRISPR finder tool, one possible CRISPR gene cluster (121 bp) was identified in the circular chromosome (starting from 778,447 and ending at 778,568) with two directed repeats (GACTGGAGCTATGACAGCAAGTCGTACAGCGA, 32 bp) and one spacer (TAACGACACGACGACCAAGACGAGCACCGAAACCAACACCGACATCAAGACCGATTAC, 57 bp). Genomic islands (GIs) prediction with IslandViewer 3 showed that there were 12 and 7 GIs in the circular and linear chromosomes, respectively (Fig. 2, for details see the web pages: http://www.pathogenomics.sfu.ca/islandviewer/accession/NZ_CP014259.1/ and http://www.pathogenomics.sfu.ca/islandviewer/accession/NZ_CP014260.1/). This is consistent with the prediction of 74 mobile genetic elements in the genome of strain S33 (20 transposases, 9 integrases and 45 conjugal transfer proteins), suggesting that lateral gene transfer has occurred frequently in the strain. In addition, 4 prophages were found in the genome of strain S33. Furthermore, there were genes encoding the plasmid partitioning protein, RepAB (AWN88_00845, 00850, 01480 and 01485) and replication initiation protein, RepC (AWN88_00855 and 01475), in the circular chromosome, despite the fact that the strain did not contain any plasmids. This is a typical feature in the smaller chromosome of *Agrobacterium* strains, indicating that the secondary chromosomes originated from an ancestral plasmid to which genes have been transferred from a progenitor primary chromosome^23^. An especially large GI in the circular chromosome (starting from 258,378 and ending at 347,893), which had a lower G + C content (56%) than that of the total chromosome (59.0%), contained the gene cluster for nicotine degradation (see next section for details).

### Gene cluster involved in nicotine degradation in strain S33, as revealed by genomic analysis

**Figure 3:**
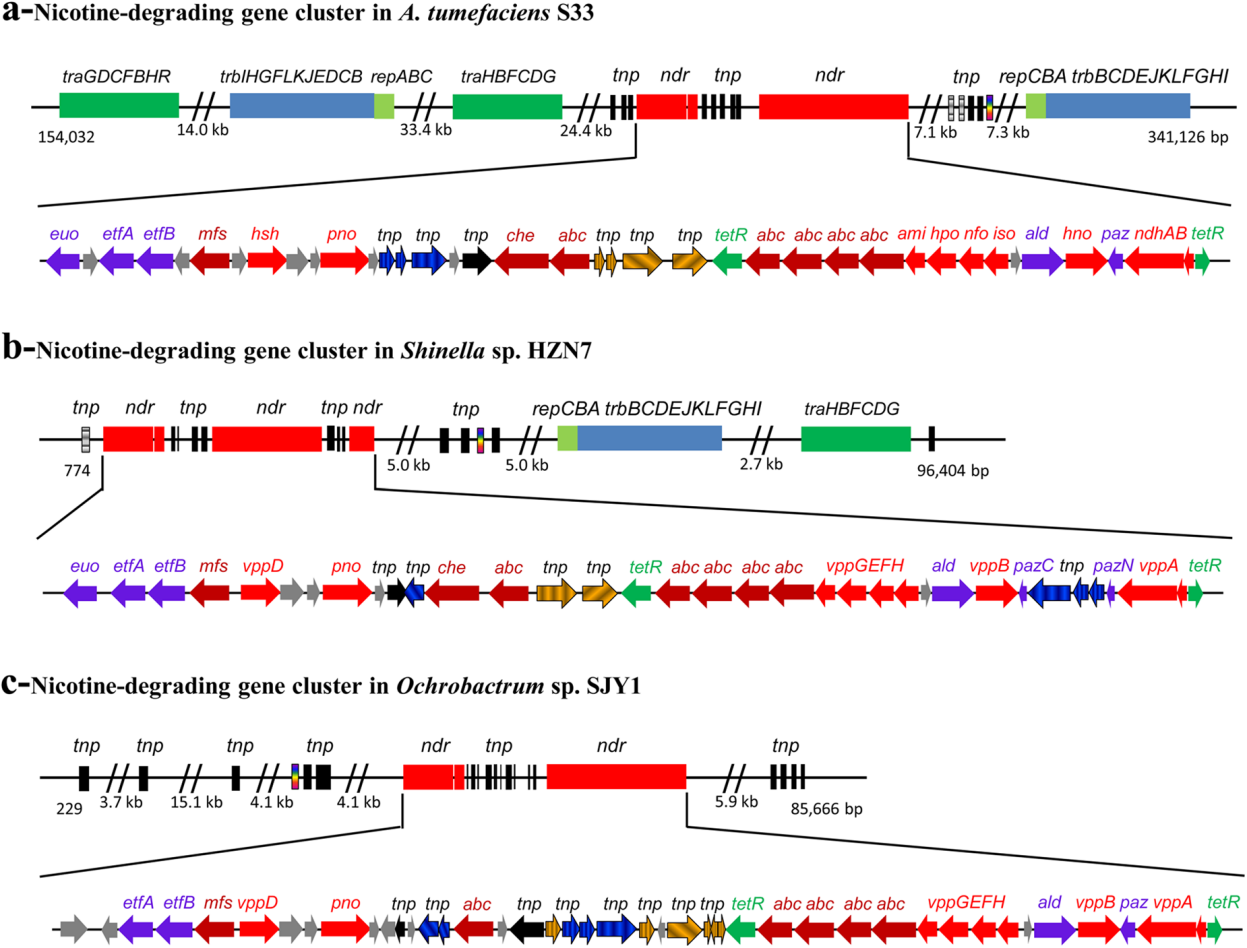
The genetic organization of the gene cluster involved in the hybrid nicotine-degrading pathway in *A*. *tumefaciens* S33, *Shinella* sp. HZN7 and *Ochrobactrum* sp. SJY1. The gene modules of *traGDCFBHR* and *trbIHGFLKJEDCB* encode conjugal transfer proteins; *repABC* encode plasmid partitioning and replication initiation proteins. *ndr*, nicotine-degrading gene cluster; *tnp*, transposase; *euo*, electron transfer flavoprotein-ubiquinone oxidoreductase; *etfAB*, electron transfer flavoprotein subunit alpha and beta; *mfs*, major facilitator superfamily transporter; *hsh* (*vppD*), 6-hydroxy-3-succinoyl-pyridine hydroxylase; *pno*, 6-hydroxypseudooxynicotine oxidase; *che*, chemotaxis protein; *abc*, ABC transporter; *tetR*, TetR family transcriptional regulator; *ami* (*vppG*), maleamate amidohydrolase (amidase); *hpo* (*vppE*), 2,5-dihydroxypyridine dioxygenase; *nfo* (*vppF*), *N*-formylmaleamate deformylase; *iso* (*vppH*), maleate cis/trans-isomerase; *ald*, aldehyde dehydrogenase; *hno* (*vppB*), 6-hydroxynicotine oxidase; *paz*, pseudoazurin; *ndhAB* (*vppA*), nicotine dehydrogenase.

Based on our previous studies, nicotine degradation in strain S33 proceeds via a hybrid pathway of the typical pyridine and pyrrolidine pathways (Fig. 1b). All the genes encoding nicotine degradation in strain S33 were located on one GI in the circular chromosome, forming a large gene cluster (Figs 2 and 3a). The enzyme initiating nicotine oxidation, nicotine dehydrogenase (NdhAB), was encoded by two ORFs (AWN88_01360 and 01355) and catalysed the hydroxylation of nicotine at the C-6 position of pyridine ring, forming 6-hydroxynicotine as the product^14^. The two subunits of the enzyme complex are similar to other hydroxylases, such as nicotinate dehydrognease (NicAB, 24.8% and 47.6% identity, respectively) from *P*. *putida* KT2440^24^, nicotine dehydrogenase (Ndh LS, 14% and 36% identity, respectively) from *A*. *nicotinovorans* ^25–27^ and isoquinoline 1-oxidoreductase (IorAB, 27% identity for both subunits) from *Brevundimonas diminuta* 7^28, 29^, where the large subunits harbour one molybdopterin cofactor, and the small subunits harbour two [2Fe2S] clusters. However, in *A*. *nicotinovorans*, the middle subunit harbouring flavin adenine dinucleotide (FAD) in Ndh is missing in strain S33, *P*. *putida* KT2440 and *B*. *diminuta* 7. Moreover, nearly identical proteins (VppA_*SL*_) are present in the nicotine-degrading bacterium *Ochrobactrum* sp. SJY1^17^. The second step in the oxidation of 6-hydroxynicotine into 6-hydroxy-*N*-methylmyosmine is catalysed by 6-hydroxynicotine oxidase (Hno, AWN88_01345) with the formation of H_2_O_2_, which binds a FAD, like other amine oxidases (W. Yu and S. Wang, unpublished data). The flavoprotein is nearly identical (99%) to the enzyme from *Shinella* sp. strain HZN7^16^ and VppB from *Ochrobactrum* sp. SJY1^18^, and it shares a 24.8% identity to the enzyme from *A*. *nicotinovorans* ^27, 30^. The third step is the non-enzymatic hydrolisation of 6-hydroxy-*N*-methylmyosmine into 6-hydroxypseudooxynicotine. The resulting 6-hydroxypseudooxynicotine is catalysed by 6-hydroxypseudooxynicotine oxidase (Pno, AWN88_01220) into 6-hydroxy-3-succinoylsemialdehyde-pyridine and methylamine in the fourth step, which binds one FMN and one [4Fe4S]^14^, and is closely related to the histamine dehydrogenase (48%) of *Pimelobacter simplex* (formerly *Nocardioides simplex*)^31–33^, the histamine dehydrogenase (46%) of *Rhizobium* sp. 4–9^34^, the trimethylamine dehydrogenase (40%) of *Methylophilus methylotrophus* W3A1^35, 36^ and the dimethylamine dehydrogenase (39%) of *Hyphomicrobium* sp. X^37^. The fifth step is oxidization of 6-hydroxy-3-succinoylsemialdehyde-pyridine into 6-hydroxy-3-succinoylpyridine. The enzyme catalysing this step has not been experimentally identified. We predict that it is encoded by AWN88_01340, which is annotated as aldehyde dehydrogenase (Ald), and has a 32% identity to the NADP^+^-dependent 3-succinoylsemialdehyde-pyridne dehydrogenase (Sapd) from *Pseudomonas* sp. HZN6^38^. The sixth step is oxidative decarboxylation of 6-hydroxy-3-succinoylpyridine to 2,5-dihydroxypyridine and succinic acid, which is catalysed by 6-hydroxy-3-succinoylpyridine hydroxylase (Hsh, AWN88_01205) in the presence of NADH^13^. The 90-kDa homodimeric flavoprotein is almost identical to the enzyme (VppD) from *Ochrobactrum* sp. SJY1^18^, and has 62% identity to the enzyme (HspB) from *Pseudomonas putida* S16^39^, as well as 17% identity to 6-hydroxynicotinate monooxygenase (NicC) from *P*. *putida* KT2440^24^. The next steps in the decomposition of 2,5-dihydroxypyridine are the same as those from the pyridine pathway for nicotine degradation in *P*. *putida* S16 (Fig. 1c) and nicotinate degradation pathway in *P*. *putida* KT2440 (Fig. 1d), and are catalysed by very similar enzymes^24, 40^. The ring opening of 2,5-dihydroxypyridine into *N*-formylmaleamic acid is likely catalysed by 2,5-dihydroxypyridine dioxygenase (Hpo, AWN88_01320), which has 81% identity to the enzyme from *P*. *putida* S16, and 42.9% to NicX from *P*. *putida* KT2440. *N*-formylmaleamic acid is then converted into maleamic and formic acid by *N*-formylmaleamate deformylase (Nfo, AWN88_01325, 62.3% identity to Nfo from strain S16; 58.2% identity to NicD from strain KT2440). The conversion of maleamic acid into maleic acid and ammonia is catalysed by maleamate amidohydrolase (Ami, AWN88_01315, 64.8% identity to Ami from strain S16; 36.7% identity to NicF from strain KT2440). Finally, maleate *cis*/*trans*-isomerase (Iso, AWN88_01330, 78% identity to Iso from strain S16; 71.6% identity to NicE from strain KT2440) catalyses the isomerization of maleic acid into fumaric acid, which then enters into the well-known Krebs cycle for further catabolism. Interestingly, nearly identical proteins (VppEFGH) are found in the nicotine-degrading bacteria *Ochrobactrum* sp. SJY1^18^ and *Shinella* sp. HZN7^41^, except that the first 55 amino acid residues at the N-terminus of Iso are missing in *Ochrobactrum* sp. SJY1. A summary of enzyme comparisons between strain S33 and those from the pyridine and pyrrolidine pathways is presented in Supplementary Table S2. Thus, nicotine, a natural *N*-heterocyclic aromatic compound and alkaloid from tobacco, is used as the sole source of carbon, nitrogen and energy to support the growth of strain S33.

All genes mentioned above that encode for nicotine degradation in strain S33 were found to form a large gene cluster with other genes encoding a transposition element and a prophage (starting at 280264 and ending at 289840), which interrupt the gene cluster into two regions: *ndhB*-*ami* part and *pno*-*hsh* part (Figs 2 and 3a). Between these two regions, there are genes encoding transposases (Tnp, AWN88_01230, 01235, 01240, 01250, 01265 and 01270), a group II intron reverse transcriptase/maturase (AWN88_01275), a DDE endonuclease (AWN88_01285), a TetR family transcriptional regulator (AWN88_01290), a chemotaxis protein (AWN88_01255) and polyamine transporters, such as putrescine/spermidine ABC transporters (AWN88_01260, AWN88_01295–01310). Since nicotine and its catabolic intermediates are amines, it is possible that these transporters and the chemotaxis protein are involved in transport during nicotine degradation.

In the context of the gene cluster, there are mainly four gene modules encoding mobile genetic elements involved in both transposition and conjugal transfer (Fig. 3a). Upstream of the gene cluster, the products of the genes are: integrases and transposases (AWN88_01165–01160, 01130 and 00875), a plasmid partitioning protein (AWN88_01070), two anti-restriction protein ArdC (AWN88_01055 and 00660), conjugal transfer proteins TraGDCFBH (AWN88_01035–01005), plasmid partitioning and replication initiation proteins RepABC (AWN88_00845–00855), conjugal transfer proteins TrbBCDEJKLFGHI (AWN88_00835–00785), a transcriptional regulator TraR(AWN88_00725), conjugal transfer proteins TraHBFACDG (AWN88_00715–00685), a nuclease (AWN88_00670) and transposases (AWN88_00585–00590, 00475 and 00400). Downstream of the gene cluster, the products of the genes are transposases (AWN88_01400–01405, 01435), a DNA invertase (AWN88_01435), an integrase (AWN88_01450), replication initiation protein RepC and plasmid partitioning protein RepAB (AWN88_01475–01485), conjugal transfer proteins TrbBCDEJKLFGHI (AWN88_01490–01540) and conjugal transfer proteins TraH (AWN88_01580). This pattern of organization suggests that the genes in the GI may have evolved due to complicated recombination and integration via transposition and conjunction.

Surprisingly, the bacteria *Shinella* sp. strain HZN7 and *Ochrobactrum* sp. strain SJY1 have highly similar nicotine-degrading genes and genetic patterns to those in strain S33^18, 41, 42^ (Fig. 3), which are not found in other strains of *A*. *tumefaciens*. The main difference is that the gene cluster in *Shinella* sp. strain HZN7 is located on a 155-kb plasmid pShin-05 (the accession number in GenBank: CP015741.1). In case of *Ochrobactrum* sp. strain SJY1, its location is not known since only the whole genome shotgun sequence is available (the accession number in GenBank for the nicotine-degrading gene cluster: KM065745). Moreover, ORFs (AWN88_01170 and AWN88_01255) encoding electron transfer flavoprotein-ubiquinone oxidoreductase (Euo) and chemotaxis protein (Che), respectively, are missing in *Ochrobactrum* sp. SJY1. And ORF (AWN88_01350) annotated to encode pseudoazurin (Paz), is split into *pazN* (encoding 63 amino acid residues at N-terminus of Paz) and *pazC* (encoding 68 amino acid residues at C-terminus of Paz) by three genes for transposase (Tnp) in *Shinella* sp. HZN7. In addition, the genetic organization and the type of the mobile element proteins around the nicotine-degrading gene clusters are distinct in the three strains (Fig. 3). There is no any gene for conjugal transfer protein around the gene cluster in *Ochrobactrum* sp. SJY1, while there are two and one sets of gene modules for conjugal transfer proteins in strain S33 and *Shinella* sp. HZN7, respectively. This suggests that the nicotine-degrading gene cluster in the plasmid pShin-05 of *Shinella* sp. HZN7 and strain S33 is transferable and could be transferred into other species via conjugation. Furthermore, the position and the number of the *tnp* genes in the gene clusters of the three strains are also different, which are consistent with the results of the insertion sequence (IS) prediction using ISfinder (http://www-is.biotoul.fr/) (see Supplementary Table S3). Although their products have different taxonomic distribution, tBlastn analysis showed that most of the *tnp* genes in the gene cluster have orthologs in the other two strains, those present relatively high similarity (30–99% identity in protein sequence, see Supplementary Table S4). However, a few of genes do not exist in one of three strains, for example, AWN88_01275 encoding group II intron reverse transcriptase/maturase is missing in strain HZN7, and AWN88_01285 encoding DDE endonuclease is missing in strain SJY1. And some genes could be fused into one or split into two genes, for example, AWN88_01265 and 01270 are fused into shn_30245 in strain HZN7. Despite high similarity in the three strains, many of these proteins showed highest similarity to the corresponding enzymes from the phylogenetically related species, which do not have any gene same or similar to the nicotine-degrading genes, as analysed using Blastp. Thus, it is difficult to set out an evolutionary trajectory of how these gene clusters came into being. For another hand, although the three strains belong to different genera, all are within the ‘*Rhizobiales* group’ of the *Alphaproteobacteria*. It is known that the same GI can occur in distantly related species as a result of various types of lateral gene transfer, such as transformation, conjugation and transposition. The fact that a highly similar GI containing the gene cluster for nicotine degradation is distributed among three different bacterial genera suggests that lateral gene transfer is responsible for the evolution of the nicotine catabolism capabilities in these Rhizobiales strains. Considering the taxonomic distribution of the mobile element proteins in the gene clusters, we assume that the nicotine-degrading gene cluster might be first transferred into a transferable plasmid such as pShin-05 of *Shinella* sp. HZN7 from *Ochrobactrum* sp. SJY1 in the way of transposition, then to other species by conjugation from the plasmid of *Shinella* sp. HZN7, where the gene cluster finally could be integrated into the chromosome of the recipient such as *A*. *tumefaciens* S33.

For the hybrid pathway of nicotine degradation in strain S33 (Fig. 1), based on chemical considerations, the first three steps from nicotine to 6-hydroxypseudooxynicotine are the same as those from the pyridine pathway of nicotine degradation identified in *A*. *nicotinovorans*. The last steps from 6-hydroxy-3-succinoylpyridine to fumaric acid are the same as those from the pyrrolidine pathway of nicotine degradation identified in *P*. *putida* S16. The process from 2,5-dihydroxypyridine to fumaric acid are the same to those from nicotinate degradation in *P*. *putida* KT2440^24^. Based on biochemical analysis, the enzymes involved in the hybrid pathway of strain S33 are functionally similar to the corresponding enzymes from the pyridine pathway in *A*. *nicotinovorans*, and the pyrrolidine pathway in *P*. *putida* S16. However, protein sequences of the enzymes for the first three steps have very low similarities to those from the pyridine pathway (see Supplementary Table S2), suggesting that the evolution of these enzymes is independent. The enzymes for the last steps from 6-hydroxy-3-succinoylpyridine to fumaric acid are highly similar to those from the pyrrolidine pathway in *P*. *putida* S16. The identities of the protein sequences are higher than those from the nicotinate-degrading enzymes in *P*. *putida* KT2440, suggesting that they are more closely related to the enzymes from the pyrrolidine pathway in the process of molecular evolution. In combination with the genetic organization of the GI gene cluster, this information could indicate that the evolution of the hybrid pathway is not a simple fusion of the pyridine and pyrollidine pathways on a molecular level, it is a result of complicated lateral gene transfer that results in a mosaic catabolic pathway, like many other ‘metabolic island’ involved in the biodegradation of pollutants^43, 44^.

### Whole transcriptome analysis

**Figure 4:**
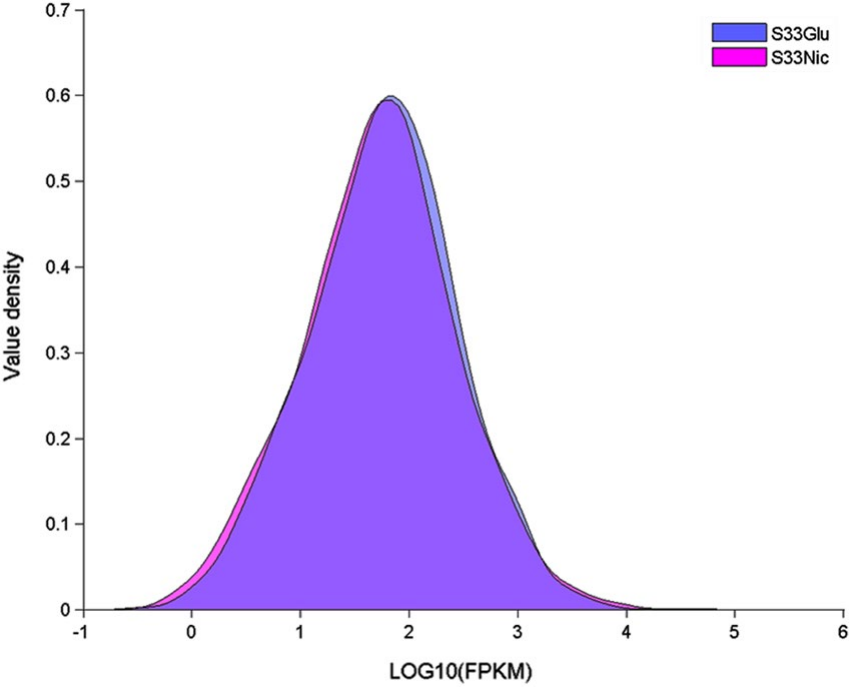
Density plot for genes expressed in *A*. *tumefaciens* S33 under two different nutrition conditions. S33Glu, cells grown in glucose-ammonium medium; S33Nic, cells grown in nicotine medium.

**Figure 5:**
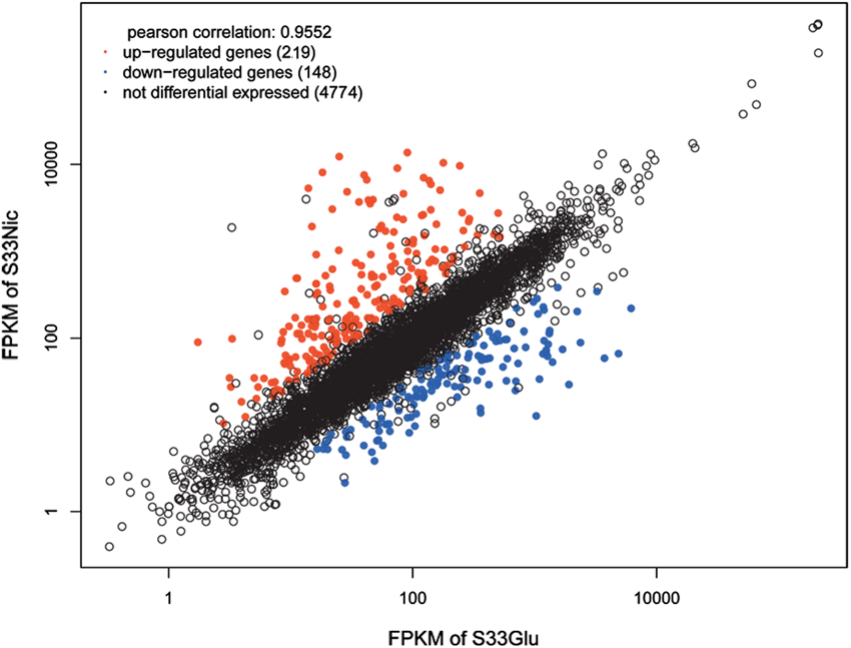
Scatter plot of FPKM values for genes expressed in *A*. *tumefaciens* S33 under two different nutrition conditions. S33Glu, cells grown in glucose-ammonium medium; S33Nic, cells grown in nicotine medium. The differentially-expressed genes were analysed based on S33Nic VS S33Glu.

**Figure 6:**
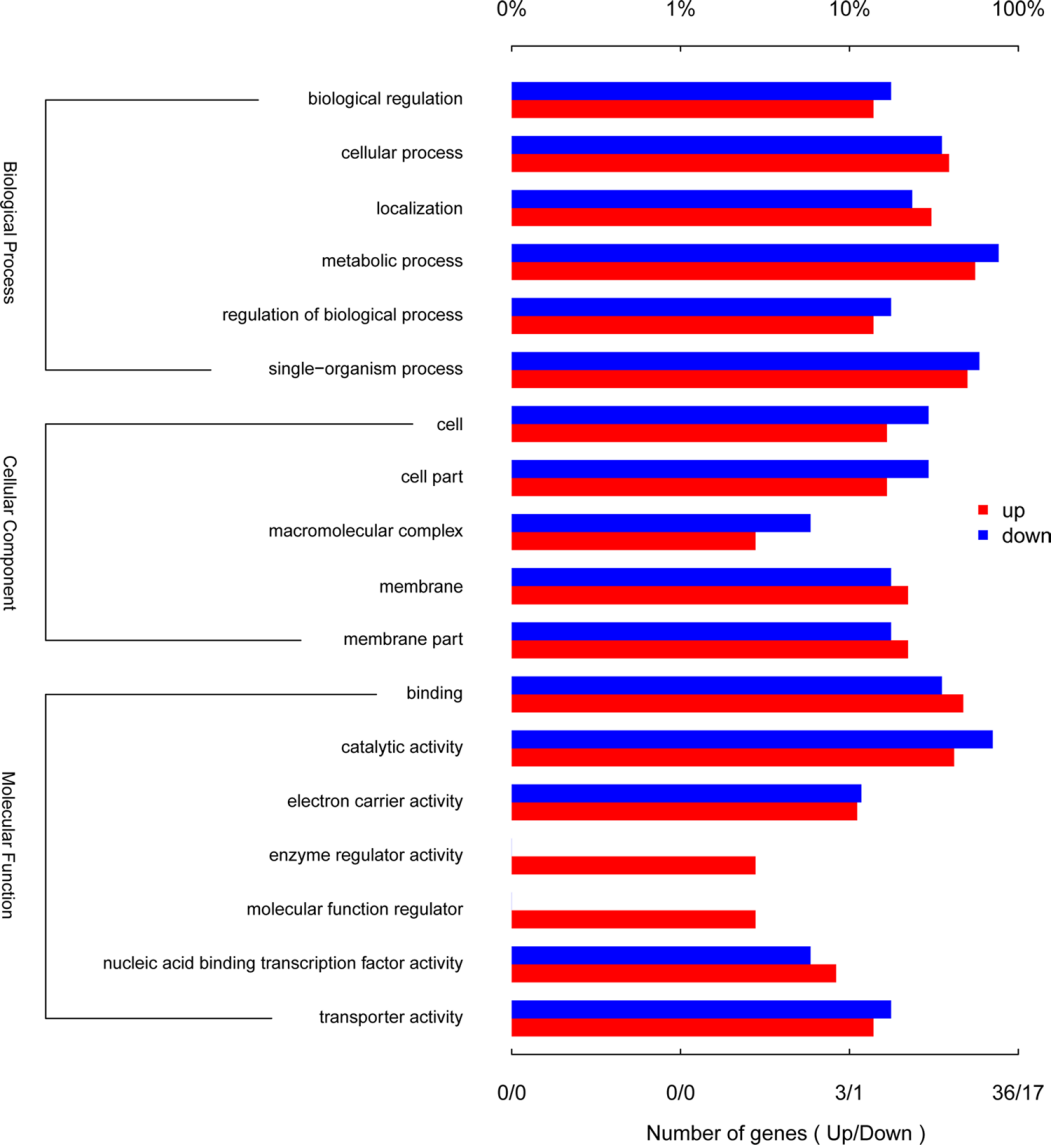
Gene ontology (GO) annotation of the differentially-expressed genes in *A*. *tumefaciens* S33 in glucose-ammonium medium (S33Glu) and nicotine medium (S33Nic). The differentially-expressed genes were analysed based on S33Nic VS S33Glu.

In order to elucidate the complete molecular mechanism of the nicotine degradation hybrid pathway, the transcriptome of strain S33 was analysed by RNA sequencing. Cells were grown under two different nutrition conditions, in glucose-ammonium medium (S33Glu) or nicotine medium with nicotine as the sole source of carbon and nitrogen (S33Nic). Raw data obtained by the Illumina Hiseq 2000 system generated 11,605,940 reads (1,163,815,055 bp) for sample S33Glu and 11,460,620 reads (1,149,073,249 bp) for sample S33Nic after filtration by quality control. The clean data were then mapped against the S33 genome and generated 11,498,956 mapped reads for S33Glu (mapped rate: 99.08%; coverage: 98%) and 11,366,729 mapped reads for S33Nic (mapped rate: 99.18%; coverage: 97%). Among the predicted 5,141 genes in the S33 genome, the numbers of genes with Fragments Per Kilobase of transcript per Million mapped reads (FPKM) ≥10 were 4,325 and 4,266 for S33Glu and S33Nic, respectively (Fig. 4). Differentially-expressed genes were identified based on gene expression-level analysis using the FPKM method with fold change (FC) values ≥2 (log_2_FC ≥ 1 or log_2_FC ≤ −1) between the two conditions. Of all 5,141 genes detected, we found 367 genes that were differentially expressed, among which, 233 genes were from the circular chromosome, and 134 genes were from the linear chromosome (see Supplementary Table S5). Of all the differentially-expressed genes, 219 genes are up-regulated, while 148 are down-regulated (S33Nic VS S33Glu) (Fig. 5). The genes in the nicotine-degrading gene cluster of the circular chromosome have the highest expression levels in the S33Nic (FPKM value is up to13,708) and highest increase in expression levels [Log_2_ Ratio (FPKM of S33Nic/FPKM of S33Glu) is up to 8.9] (Table 1). The gene ontology (GO) annotation summary of differentially-expressed genes is shown in Fig. 6.

**Table 1:** Differently up-regulated genes in the nicotine-degrading gene cluster when *A*. *tumefaciens* S33 grows on nicotine compared to on glucose and ammonium sulfate.

Locus tag	Gene function	Fpkm_value _S33Glu	Fpkm_value _S33Nic	Log_2_ Ratio(Nic/Glu)	*p*_value
AWN88_01170	electron transfer flavoprotein-ubiquinone oxidoreductase	16.5721	331.022	4.3201	0.00005
AWN88_01175	hypothetical protein	11.3119	485.983	5.42499	0.0002
AWN88_01180	electron transfer flavoprotein subunit alpha	44.7696	3518.5	6.2963	0.00015
AWN88_01185	electron transfer flavoprotein subunit beta	47.2122	3916.53	6.37427	0.0002
AWN88_01190	hypothetical protein	243.228	9641.61	5.30889	0.00005
AWN88_01195	MFS transporter	90.4225	13708.9	7.24422	0.0328
AWN88_01205	Hsh, 6-hydroxy-3-succinoyl-pyridine hydroxylase	41.9982	6642.84	7.30533	0.00205
AWN88_01210	hypothetical protein	45.6245	3827.51	6.39045	0.00955
AWN88_01215	hypothetical protein	136.334	6502.5	5.57577	0.00095
AWN88_01220	Pno, 6-hydroxypseudooxynicotine oxidase	121.913	2627.11	4.42956	0.02195
AWN88_01225	hypothetical protein	12.2644	139.284	3.50547	0.0003
AWN88_01255	chemotaxis protein	48.4621	506.969	3.38697	0.00125
AWN88_01260	putrescine/spermidine ABC transporter substrate-binding protein	122.901	7038.91	5.83978	0.00555
AWN88_01295	putrescine/spermidine ABC transporter substrate-binding protein	141.015	5902.87	5.3875	0.0128
AWN88_01310	spermidine/putrescine ABC transporter ATP-binding protein	43.2709	3875.89	6.48499	0.00825
AWN88_01315	Ami, maleamate amidase	28.9885	4835.76	7.38212	0.00005
AWN88_01320	Hpo, 2,5-dihydroxypyridine dioxygenase	24.9847	12309.7	8.94454	0.03205
AWN88_01325	Nfo, *N*-formylmaleamate deformylase	13.9544	5307.77	8.57124	0.00245
AWN88_01330	Iso, maleate isomerase	18.2106	8084.81	8.79429	0.00095
AWN88_01335	hypothetical protein	39.9672	7550.02	7.56152	0.00015
AWN88_01340	Ald, aldehyde dehydrogenase	74.8364	9053.21	6.91855	0.03385
AWN88_01345	Hno, 6-hydroxynicotine oxidase	178.319	10441.2	5.87168	0.0317
AWN88_01350	Paz, pseudoazurin	167.75	5045.96	4.91074	0.00275
AWN88_01355–01360	NdhAB, nicotine dehydrogenase	21.9143	3044.96	7.11841	0.00045
AWN88_01365	TetR family transcriptional regulator	21.1065	354.632	4.07056	0.00015

### Transcriptional analysis of nicotine-degrading genes in strain S33

**Figure 7:**
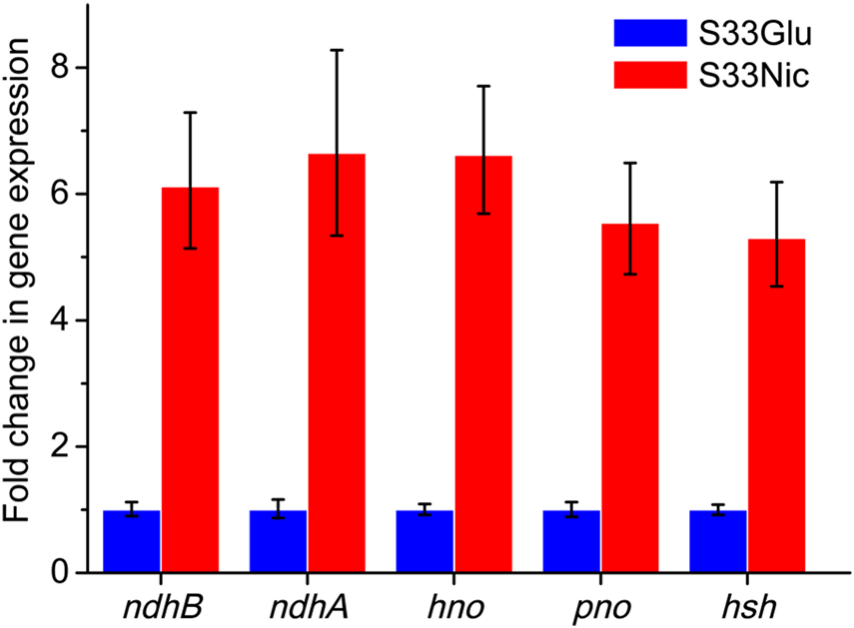
qRT-PCR analysis of the expression levels of five genes involved in nicotine degradation in *A*. *tumefaciens* S33. S33Glu, cells grown in glucose-ammonium medium; S33Nic, cells grown in nicotine medium. The expression levels in glucose-ammonium medium were used as control and set as 1.

The 9 nicotine-degrading genes (*hsh*, *pno*, *ami*, *hpo*, *nfo*, *iso*, *hno* and *ndhAB*) that have been experimentally verified in S33, *Shinella* sp. strain HZN7, or *Ochrobactrum* sp. strain SJY1, have the highest expression levels in the S33Nic (FPKM value is at least around 3,000). In contrast, their expression levels in the S33Glu are very low (FPKM value is less than 200) (Table 1). These genes exhibited a significant increase in transcriptional activities in nicotine medium compared to those in glucose-ammonium medium, as measured by the Log_2_ Ratio (FPKM of S33Nic/FPKM of S33Glu), ranging from 4.4 to 8.9. This indicates that the genes play key roles in nicotine catabolism by strain S33, and their transcriptional activities required the presence of nicotine. Results were confirmed by real-time quantitative reverse transcription PCR (qRT-PCR) analysis of the expression levels of five genes (*ndhA*, *ndhB*, *hno*, *pno* and *hsh*) (Fig. 7), which also validates the reliability of RNA-seq data in this study.

### Prediction of genes potentially involved in nicotine degradation, based on transcriptional analysis

Along with the nine genes in the nicotine-degrading gene cluster that directly catalysing nicotine degradation, 11 additional genes in the same gene cluster have much higher expression levels in nicotine medium than in glucose-ammonium medium (Table 1 and Fig. 3a). Functional analysis also predicted that these genes were involved in nicotine catabolism. The ORF AWN88_01340, which is located between *iso* and *hno*, is annotated as aldehyde dehydrogenase (Ald) and is expected to catalyse the fifth step of the pathway, oxidization of 6-hydroxy-3-succinoylsemialdehyde-pyridne into 6-hydroxy-3-succinoyl-pyridine. It shares 32% identity to the NADP^+^-dependent 3-succinoylsemialdehyde-pyridne dehydrogenase (Sapd) from *Pseudomonas* sp. HZN6^38^. In addition, four genes are annotated to encode putrescine/spermidine ABC transporters (Abc, AWN88_01260, AWN88_01295, AWN88_01310) and major facilitator superfamily transporter (Mfs, AWN88_01195). They may function in substrate transport during nicotine degradation. Correlatively, the chemotaxis protein gene (Che, AWN88_01255) directly in the upstream of the ABC transporter genes may be responsible for substrate sensing. Furthermore, another four genes are predicted to be involved in electron transfer and energy production during nicotine oxidation. The ORF (AWN88_01350) is annotated to encode pseudozaurin, a small copper-containing electron carrier, which shares 63% and 38% identity with the protein from the denitrifiers *Sinorhizobium meliloti* ^45^ and *Paracoccus pantotrophus* (formerly *Thiosphaera pantotropha*)^46, 47^, respectively. It is well known that pseudoazurin functions as an electron donor inside the periplasmic space for several enzymes in the denitrification process, including nitrite reductase, nitrous oxide reductase and nitric oxide reductase^46^. Since it is one of the differently-expressed genes with Log_2_ Ratio (FPKM of S33Nic/ FPKM of S33Glu) of 4.9 in nicotine medium versus glucose-ammonium medium, and is directly located in the upstream of *ndhAB*, it is expected to function as the electron acceptor of nicotine dehydrogenase. This activity was determined using an artificial electron acceptor, such as 2,6-dichlorophenolindophenol (DCIP)^14^. The ORFs (AWN88_01180 and AWN88_01185) are predicted to encode electron transfer flavoprotein subunits alpha and beta (EtfAB), which may pick up electrons from nicotine oxidation, and then transfer them to ubiquinone. This reaction is catalysed by the electron transfer flavoprotein-ubiquinone oxidoreductase (Euo), which is encoded by ORF (AWN88_01170). Thus, the electrons enter the respiration chain and finally are accepted by O_2_; however, the manner in which they function in the process of nicotine oxidation, and how the electrons are transferred to O_2_ via the respiration chain will require further experimental verification. Moreover, there is a TetR family transcriptional regulator gene (AWN88_01365) adjacent to *ndhAB* in the gene cluster that shares 36% identity with the TetR family member NicR2 from *P*. *putida* S16^48^ and potentially may be involved in the regulation of nicotine degradation, despite its opposite transcriptional direction to *ndhAB* (Fig. 3a).

## Conclusion

*A*. *tumefaciens* S33 degrades nicotine via a novel hybrid pathway of the traditional pyridine and pyrrolidine pathways. In this study, we explored the molecular mechanism of this novel pathway by analysing its complete genome and transcriptomes using both glucose-ammonium medium and nicotine medium for growth. Interestingly, the nicotine-degrading genes, together with genes encoding the plasmid partitioning protein, plasmid replication initiation protein, conjugal transfer proteins and transposases, form a large gene cluster that is located on a GI in the circular chromosome of strain S33. Protein sequence analysis showed that the products of the nicotine-degrading genes have low similarity to the corresponding enzymes from the pyridine and pyrrolidine pathways, although they catalyse the same reactions. This indicates that the evolution of the novel hybrid pathway of nicotine degradation in strain S33 is not a simple fusion of the genes involved in the two pathways, but instead, the result of complicated lateral gene transfer. Moreover, a highly similar gene cluster exists in two other phylogenetically similar bacteria, *Ochrobactrum* sp. SJY1 and *Shinella* sp. strain HZN7 from the ‘*Rhizobiales* group’ of the *Alphaproteobacteria*. In addition, based on comparative transcriptome analysis and previous knowledge, we predicted that some genes in this gene cluster are potentially involved in substrate sensing and transport, transcription regulation and electron transfer during nicotine degradation. Thus, the whole system for nicotine catabolism via the hybrid pathway is outlined. These results provide new clues for further elucidating the molecular mechanism of this novel hybrid nicotine degradation pathway. Our efforts in the future will be put towards verifying our predictions and identifying the functions of the genes in this novel hybrid pathway.

## Methods

### Strains and growths

*Agrobacterium tumefaciens* S33, was deposited at China Center for Type Culture Collection under the accession number CCTCC AB 2016054 (originally CCTCC M 206131). It was grown in nicotine medium with nicotine (1.0 g l^−1^) as the sole source of nitrogen and carbon, or glucose-ammonium medium containing 1.0 g l^−1^ glucose, 0.2 g l^−1^ ammonium sulphate, and 1.0 g l^−1^ yeast extract at 30 °C, as described previously^2, 13^.

### Gene prediction and annotation

The genome sequence of the strain S33 was determined using the PacBio sequencing technology^19^. Gene prediction and genome annotation were performed using the NCBI PGAP version 3.1 (http://www.ncbi.nlm.nih.gov/genome/annotation_prok/) and the Rapid Annotations using Subsystems Technology server^49^. The locus tag prefix was set as ‘AWN88’. For sequence alignments, the software of Vector NTI Advance 11 (Invitrogen) and the Blast program of NCBI were used. CRISPR finder (http://crispr.u-psud.fr/Server/) was used for identifying CRISPR systems^50^. GIs were predicted by IslandViewer 3 (http://www.pathogenomics.sfu.ca/islandviewer), which integrates three different genomic island prediction methods: IslandPick, IslandPath-DIMOB, and SIGI-HMM^51, 52^.

### Transcriptome sequencing and analysis

*A*. *tumefaciens* S33 was grown in nicotine medium or glucose-ammonium medium and harvested at the early-exponential phase. For RNA sequencing, cells were immediately frozen by liquid nitrogen after being harvested. The RNA was extracted with TRIzol Reagent (Invitrogen) and sequenced by using the Illumina Hiseq 2000 system (Majorbio, Shanghai, China). Before sequencing, the rRNA in the sample was removed by the Ribo-Zero Magnetic kit (G^+^/G^−^ Bacteria) (EpiCentre). Then the purified mRNA was used for library construction with Illumina Truseq RNA sample prep Kit according to the manufacturer’s protocol. After amplification by PCR and recovery by electrophoresis with 2% Certified Low Range Ultra Agarose (Bio-Rad), the sample was further amplified by bridge PCR with Illumina Truseq PE Cluster Kit v3-cBot-HS on cBot (Illumina). Finally, samples were sequenced using Hiseq2000 Truseq SBS Kit v3-HS (200 cycles) (Illumina) with the read length as 2 × 100 (PE100). Raw sequencing reads were subjected to filtration by quality control, and then mapped against the genome of S33. The Majorbio (Shanghai, China) performed further analysis, as previously described^53^. Differentially-expressed genes were identified by gene expression-level analysis using the FPKM (Fragments Per Kilobase of transcript per Million mapped reads) method with *p* values ≤ 0.05 and fold change (FC) values ≥2 (log2 FC ≥ 1 or log2 FC ≤ −1)^54, 55^. The Cuffdiff program (http://cufflinks.cbcb.umd.edu/) was used to analyse differences between these two conditions.

### Real-time quantitative reverse transcription PCR (qRT-PCR)

To confirm the results of RNA sequencing, five genes (*ndhA*, *ndhB*, *hno*, *pno* and *hsh*) were chosen for qRT-PCR analysis. Cells were harvested at the early-exponential phase and total RNA was extracted using Ezol Total RNA Extraction Reagent (Shanghai GenePharma, China) according to the manufacturer’s protocol. During this process, DNA was removed by digestion with DNaseI. Total cDNA was synthesized with TransScript First-Strand cDNA Synthesis SuperMix (Beijing TransGen Biotech, China). qPCR was performed by using SYBR Real-Time PCR Master Mix (Shanghai GenePharma, China) according to the manufacturer’s protocol. The 16S rRNA gene was used as the reference gene. Reactions were performed in triplicate. The primers employed in amplification are listed in the Supplementary Table S1.

## Electronic supplementary material

supplemental materials

## References

[1] Brandsch R (2006). Microbiology and biochemistry of nicotine degradation. Appl. Microbiol. Biotechnol..

[2] Wang S, Huang H, Xie K, Xu P (2012). Identification of nicotine biotransformation intermediates by *Agrobacterium tumefaciens* strain S33 suggests a novel nicotine degradation pathway. Appl. Microbiol. Biotechnol..

[3] Liu J (2015). Nicotine-degrading microorganisms and their potential applications. Appl. Microbiol. Biotechnol..

[4] Civilini M, Domenis C, Sebastianutto N, de Berfoldi M (1997). Nicotine decontamination of tobacco agro-industrial waste and its degradation by micro-organisms. Waste Manag. Res..

[5] Enamorado MF, Ondachi PW, Comins DL (2010). A five-step synthesis of (*S*)-macrostomine from (*S*)-nicotine. Org. Lett..

[6] Roduit JP, Wellig A, Kiener A (1997). Renewable functionalized pyridines derived from microbial metabolites of the alkaloid (*S*)-nicotine. Heterocycles.

[7] Schmid A (2001). Industrial biocatalysis today and tomorrow. Nature.

[8] Wang SN (2005). “Green” route to 6-hydroxy-3-succinoyl-pyridine from (*S*)-nicotine of tobacco waste by whole cells of a *Pseudomonas* sp. Environ. Sci. Technol..

[9] Wang W, Xu P, Tang H (2015). Sustainable production of valuable compound 3-succinoyl-pyridine by genetically engineering *Pseudomonas putida* using the tobacco waste. Sci. Rep..

[10] Yu H, Tang H, Xu P (2014). Green strategy from waste to value-added-chemical production: efficient biosynthesis of 6-hydroxy-3-succinoyl-pyridine by an engineered biocatalyst. Sci. Rep..

[11] Tang H (2013). Systematic unraveling of the unsolved pathway of nicotine degradation in *Pseudomonas*. PLoS genetics.

[12] Wang SN, Liu Z, Xu P (2009). Biodegradation of nicotine by a newly isolated *Agrobacterium* sp. strain S33. J. Appl. Microbiol..

[13] Li H, Xie K, Huang H, Wang S (2014). 6-hydroxy-3-succinoylpyridine hydroxylase catalyzes a central step of nicotine degradation in *Agrobacterium tumefaciens* S33. PloS one.

[14] Li H (2016). Nicotine dehydrogenase complexed with 6-hydroxypseudooxynicotine oxidase involved in the hybrid nicotine-degrading pathway in *Agrobacterium tumefaciens* S33. Appl. Environ. Microbiol..

[15] Ma Y (2014). Isolation, transposon mutagenesis, and characterization of the novel nicotine-degrading strain *Shinella* sp. HZN7. Appl. Microbiol. Biotechnol..

[16] Qiu J (2014). A novel (*S*)-6-hydroxynicotine oxidase gene from *Shinella* sp. strain HZN7. Appl. Environ. Microbiol..

[17] Yu H, Tang H, Li Y, Xu P (2015). Molybdenum-containing nicotine hydroxylase genes in a nicotine degradation pathway that is a variant of the pyridine and pyrrolidine pathways. Appl. Environ. Microbiol..

[18] Yu H, Tang H, Zhu X, Li Y, Xu P (2015). Molecular mechanism of nicotine degradation by a newly isolated strain, *Ochrobactrum* sp. strain SJY1. Appl. Environ. Microbiol..

[19] Yu W (2016). Genome sequence of the nicotine-degrading *Agrobacterium tumefaciens* S33. J. Biotechnol..

[20] Goodner B (2001). Genome sequence of the plant pathogen and biotechnology agent *Agrobacterium tumefaciens* C58. Science.

[21] Huang, Y. Y. *et al*. Complete genome sequence of *Agrobacterium tumefaciens* Ach5. *Genome Announc*. **3**, doi:10.1128/genomeA.00570-15 (2015). 10.1128/genomeA.00570-15 PMC4457062 26044425

[22] Mondy, S., Lalouche, O., Dessaux, Y. & Faure, D. Genome sequence of the quorum-sensing-signal-producing nonpathogen *Agrobacterium tumefaciens* strain P4. *Genome Announc*. **1**, doi:10.1128/genomeA.00798-13 (2013). 10.1128/genomeA.00798-13 PMC3790094 24092790

[23] Slater SC (2009). Genome sequences of three agrobacterium biovars help elucidate the evolution of multichromosome genomes in bacteria. J. Bacteriol..

[24] Jiménez JI (2008). Deciphering the genetic determinants for aerobic nicotinic acid degradation: the nic cluster from Pseudomonas putida KT2440. Proc. Natl. Acad. Sci. USA.

[25] Freudenberg W, König K, Andreesen JR (1988). Nicotine dehydrogenase from *Arthrobacter oxidans*: A molybdenum-containing hydroxylase. FEMS Microbiol. Lett..

[26] Grether-Beck S (1994). Structural analysis and molybdenum-dependent expression of the pAO1-encoded nicotine dehydrogenase genes of *Arthrobacter nicotinovorans*. Mol. Microbiol..

[27] Igloi GL, Brandsch R (2003). Sequence of the 165-kilobase catabolic plasmid pAO1 from *Arthrobacter nicotinovorans* and identification of a pAO1-dependent nicotine uptake system. J. Bacteriol..

[28] Lehmann M, Tshisuaka B, Fetzner S, Lingens F (1995). Molecular cloning of the isoquinoline 1-oxidoreductase genes from *Pseudomonas diminuta* 7, structural analysis of iorA and iorB, and sequence comparisons with other molybdenum-containing hydroxylases. J. Biol. Chem..

[29] Lehmann M, Tshisuaka B, Fetzner S, Roger P, Lingens F (1994). Purification and characterization of isoquinoline 1-oxidoreductase from *Pseudomonas diminuta* 7, a novel molybdenum-containing hydroxylase. J. Biol. Chem..

[30] Dai VD, Decker K, Sund H (1968). Purification and properties of L-6-hydroxynicotine oxidase. Eur. J. Biochem..

[31] Fujieda N, Satoh A, Tsuse N, Kano K, Ikeda T (2004). 6-S-cysteinyl flavin mononucleotide-containing histamine dehydrogenase from *Nocardioides simplex*: molecular cloning, sequencing, overexpression, and characterization of redox centers of enzyme. Biochemistry.

[32] Limburg J, Mure M, Klinman JP (2005). Cloning and characterization of histamine dehydrogenase from *Nocardioides simplex*. Arch. Biochem. Biophys..

[33] Reed T (2010). Crystal structure of histamine dehydrogenase from *Nocardioides simplex*. J. Biol. Chem..

[34] Bakke M, Sato T, Ichikawa K, Nishimura I (2005). Histamine dehydrogenase from *Rhizobium* sp.: gene cloning, expression in *Escherichia coli*, characterization and application to histamine determination. J. Biotechnol..

[35] Boyd G, Mathews FS, Packman LC, Scrutton NS (1992). Trimethylamine dehydrogenase of bacterium W3A1. Molecular cloning, sequence determination and over-expression of the gene. FEBS Lett..

[36] Lim LW (1986). Three-dimensional structure of the iron-sulfur flavoprotein trimethylamine dehydrogenase at 2.4-A resolution. J. Biol. Chem..

[37] Yang CC, Packman LC, Scrutton NS (1995). The primary structure of *Hyphomicrobium X* dimethylamine dehydrogenase. Relationship to trimethylamine dehydrogenase and implications for substrate recognition. Eur. J. Biochem..

[38] Qiu J (2012). Functional identification of two novel genes from *Pseudomonas* sp. strain HZN6 involved in the catabolism of nicotine. Appl. Environ. Microbiol..

[39] Tang H (2011). A novel NADH-dependent and FAD-containing hydroxylase is crucial for nicotine degradation by *Pseudomonas putida*. J. Biol. Chem..

[40] Tang H (2012). Genomic analysis of *Pseudomonas putida*: genes in a genome island are crucial for nicotine degradation. Sci. Rep.

[41] Qiu J (2016). The complete genome sequence of the nicotine-degrading bacterium *Shinella* sp. HZN7. Fron. Microbiol..

[42] Yu, H., Li, Y., Tang, H. & Xu, P. Genome sequence of a newly isolated nicotine-degrading bacterium, *Ochrobactrum* sp. SJY1. *Genome Announc*. **2**, doi:10.1128/genomeA.00720-14 (2014). 10.1128/genomeA.00720-14 PMC4110227 25059869

[43] Juhas M (2009). Genomic islands: tools of bacterial horizontal gene transfer and evolution. FEMS Microbiol. Rev..

[44] Fulthorpe, R. R. & Top, E. M. In *Handbook of Hydrocarbon and Lipid Microbiology* (ed Timmis, K. N.) 1219–1229 (Springer-Verlag Berlin Heidelberg, 2010).

[45] Laming EM, McGrath AP, Guss JM, Kappler U, Maher MJ (2012). The X-ray crystal structure of a pseudoazurin from *Sinorhizobium meliloti*. J. Inorg. Biochem..

[46] Najmudin S, Pauleta SR, Moura I, Romao MJ (2010). The 1.4 A resolution structure of *Paracoccus pantotrophus* pseudoazurin. Acta Crystallog. Sect. F Struct. Biol. Cryst. Commun..

[47] Leung YC (1997). The pseudoazurin gene from *Thiosphaera pantotropha*: analysis of upstream putative regulatory sequences and overexpression in *Escherichia coli*. Biochem. J..

[48] Wang L, Tang H, Yu H, Yao Y, Xu P (2014). An unusual repressor controls the expression of a crucial nicotine-degrading gene cluster in *Pseudomonas putida* S16. Mol. Microbiol..

[49] Aziz R (2008). The RAST Server: rapid annotations using subsystems technology. BMC Genomics.

[50] Grissa I, Vergnaud G, Pourcel C (2007). CRISPRFinder: a web tool to identify clustered regularly interspaced short palindromic repeats. Nucleic Acids Res..

[51] Dhillon BK (2015). IslandViewer 3: more flexible, interactive genomic island discovery, visualization and analysis. Nucleic Acids Res..

[52] Langille MG, Hsiao WW, Brinkman FS (2010). Detecting genomic islands using bioinformatics approaches. Nat. Rev. Microbiol..

[53] Wang Y (2013). Comparative transcriptome analysis of tomato (Solanum lycopersicum) in response to exogenous abscisic acid. BMC Genomics.

[54] Trapnell C (2013). Differential analysis of gene regulation at transcript resolution with RNA-seq. Nat. Biotechnol..

[55] Trapnell C (2010). Transcript assembly and quantification by RNA-Seq reveals unannotated transcripts and isoform switching during cell differentiation. Nat. Biotechnol..

