# Supplementary material for: Genomic and transcriptomic analyses of Agrobacterium tumefaciens S33 reveal the molecular mechanism of a novel hybrid nicotine-degrading pathway
Source: Sci Rep. 2017 Jul 6;7:4813. doi: 10.1038/s41598-017-05320-1 (PMC5500553; doi:10.1038/s41598-017-05320-1)
Supplement: Supplementary file 1 — supplemental materials [file 41598_2017_5320_MOESM1_ESM.pdf]

Supplementary information

Genomic and transcriptomic analyses of *Agrobacterium tumefaciens* S33 reveal the molecular mechanism of a novel hybrid nicotine-degrading pathway

Haiyan Huang [1,2], Wenjun Yu [1], Rongshui Wang[1], Huili Li [1], Huijun Xie [3] & Shuning Wang [1,]*

[1]State Key Laboratory of Microbial Technology, School of life science, Shandong University, Jinan 250100, People's Republic of China, [2]Institute of Basic Medicine, Shandong Academy of Medical Science, Jinan 250062, People's Republic of China, [3]Environment Research Institute, Shandong University, Jinan 250100, People's Republic of China

* Correspondence and requests for materials should be addressed to S.W. (email: shuningwang@sdu.edu.cn)

Table S1. The primers used for qRT-PCR.

Primers	Sequence (5′-3′)	Length of the amplified fragment (bp)
16S-F	ACTCTGGAACTGCCTTTGATA	
16S-R	CGTTTACGGCGTGGACTA	197
ndhA-F	AGTCATTGGAGATTGAGTGG	
ndhA-R	GCTTCTTTGATCCTTTTGC	142
ndhB-F	AATGCGGCTATTGCCAGTC	
ndhB-R	GCGGATGCGAGGATAAGTG	134
hno-F	TTGCGGATAAGAGGGTGT	
hno-R	GGTGGTAGTGGTGGTTCG	94
pno-F	CGGTAGAAGACAGGCGACG	
pno-R	CCACGGCCAATCACTAACAT	119
hsh-F	GCGTGACATTTATGGAAGACCC	
hsh-R	GCGAAAATGCCTCCACCTC	117

Table S2. Comparison of protein sequences of key enzymes involved in nicotine degradation in *A. tumefaciens* S33 and functionally similar proteins from other nicotine/nicotinate-degrading bacteria. [a, b]

***A. tumefaciens* S33** (hybrid)	**Number of amino acids**	***Ochrobactrum* sp. SJY1** (hybrid)	***Shinella* sp. HZN7** (hybrid)	***P. putida* S16** (pyrrolidine)	***Pseudomonas* sp. HZN6** (pyrrolidine)	***A. nicotinovorans*** (pyridine)	***P. putida* KT2440** (nicotinate)
NdhB (AWN88_01360)	155	100% (VppAs)	100%	34.9% (SpmBC)		36.4% (NdhS) 32.3% (KdhS)	47.6% (NicA)
NdhA (AWN88_01355)	749	99.6% (VppAL)	99.6%	6.4% (SpmA)		14% (NdhL) 14.4% (KdhL)	24.8% (NicB)
Hno (AWN88_01345)	437	99.8% (VppB)	99.8% (NctB)	38.4% (NicA2)	38.9% (Nox)	24.8% (Hno)	
Pno (AWN88_01220)	671	100%	100%	2.9% (Pnao)	11.0% (Pnao)	5.2% (NdhM) 4.6% (KdhM)	
Ald (AWN88_01340)	465	99.8%	99.8%	35.5% (Sapd)	32.2% (Sapd)		
Hsh (AWN88_01205)	391	99.7% (VppD)	99.7%	62.7% (HspB)			17% (NicC)
Iso (AWN88_01330)	249	100% (VppH)	100%	78.0% (Iso)			71.6% (NicE)
Nfo (AWN88_01325)	260	99.6% (VppF)	99.6%	62.3% (Nfo)			58.2% (NicD)
Hpo (AWN88_01320)	342	100% (VppE)	99.7%	81.0% (Hpo)			42.9% (NicX)
Ami (AWN88_01315)	210	99.5% (VppG)	99.5%	64.8% (Ami)			36.7% (NicF)

a, identity of protein sequences analyzed by Blast is shown;

b, the type of pathway is annotated in the parentheses behind the name of bacteria, where, hybrid, the hybrid pathway; pyrrolidine, the pyridine pathway; pyridine, the pyridine pathway; nicotinate, nicotinate-degrading pathway.

Table S3. Insertion sequence (IS) annotation in the nicotine-degrading gene clusters of *A. tumefaciens* S33, *Shinella* sp. HZN7 and *Ochrobactrum* sp. SJY1 using ISfinder (http://www-is.biotoul.fr/).

Range	Length (bp)	IS family	Gene Products
***A. tumefaciens* S33**			
193814_191431	2383	IS3_ssgr_IS51	AWN88_00855-00860
259648_257301	2347	IS630	AWN88_01125-01135
261897_264995	3098	IS3_ssgr_IS407	AWN88_01155-01170
274940_279283	4343	IS66	AWN88_01220-01250
277837_280987	3150	ISL3	AWN88_01240-01255
277284_279588	2304	IS66	AWN88_01240-01255
283927_286677	2750	IS3_ssgr_IS51	AWN88_01260-01275
287332_290839	3507	IS630	AWN88_01275-01290
286902_289131	2229	IS3_ssgr_IS51	AWN88_01275-01290
314045_311662	2383	IS630	AWN88_01395-01405
313467_311102	2365	IS630	AWN88_01395-01405
***Shinella* sp. HZN7**			
1_2135	2134	IS630	shn_30135, RS35330, 30150
292_2719	2427	IS630	shn_30135-30150, RS35330, RS35335
4338_1484	2854	IS630	shn_30160, RS35335, 30150, RS35330
17684_20396	2712	IS110_ssgr_IS1111	shn_30205-30220
21947_18809	3138	IS3_ssgr_IS150	shn_30230, 30220, 30215
24600_27635	3035	IS3_ssgr_IS51	shn_30235-shn_30250
44467_40012	4455	IS66	shn_30305-shn_30325
55137_50809	4328	IS21	shn_30360-shn_30385
64147_59842	4305	IS21	shn_30405- shn_30430
94382_97403	3021	IS110_ssgr_IS1111	shn_RS35360, shn_30590-shn_30595

Ochrobactrum sp. SJY1			
1_3444	3443	Tn3	AIH15730.1, AIH15731.1
8725_5187	3538	IS3_ssgr_IS150	AIH15735.1, AIH15734.1, AIH15733.1
24967_21876	3091	IS5_ssgr_IS5	AIH15748.1, AIH15749.1, AIH15750.1, AIH15751.1
33961_28995	4966	Tn3	AIH15755.1, AIH15756.1, AIH15757.1, AIH15758.1, AIH15759.1
51811_48832	2979	IS66	AIH15773.1, AIH15774.1, AIH15775.1, AIH15776.1, AIH15777.1, AIH15778.1
53091_49953	3138	IS3_ssgr_IS150	AIH15775.1, AIH15776.1, AIH15777.1, AIH15778.1, AIH15779.1, AIH15780.1
54967_59421	4454	IS66	AIH15782.1, AIH15783.1, AIH15784.1, AIH15785.1, AIH15786.1, AIH15787.1, AIH15789.1
54494_56822	2328	IS3_ssgr_IS51	AIH15782.1, AIH15783.1, AIH15784.1, AIH15785.1
56351_53155	3196	IS256	AIH15780.1, AIH15781.1, AIH15782.1, AIH15783.1, AIH15784.1
57510_59811	2301	IS3_ssgr_IS51	AIH15786.1, AIH15787.1, AIH15789.1
60058_62512	2454	IS3_ssgr_IS51	AIH15789.1, AIH15790.1, AIH15791.1, AIH15792.1, AIH15793.1
83540_86171	2631	IS630	AIH15815.1, AIH15816.1, AIH15817.1, AIH15818.1
84292_86871	2579	IS3_ssgr_IS150	AIH15815.1, AIH15816.1, AIH15817.1, AIH15818.1, AIH15819.1

Table S4. Transposases from the nicotine-degrading gene clusters of *A. tumefaciens* S33, *Shinella* sp. HZN7 and *Ochrobactrum* sp. SJY1. The proteins were analysed by using tBlastn and PFAM database (http://pfam.xfam.org/).

Locus tag in GenBank or position in the gene cluster	Protein_id	Gene function	Size (amino acids)	Description	tBlastn against the gene cluster from other two strains (%, identity)
A. tumefaciens S33					
AWN88_01130		transposase		Pseudo, incomplete, miss stop	
AWN88_01160	AMD56917.1	transposase	88	HTH_Tnp_1, Transposase, pfam01527	
AWN88_01165	AMD56918.1	integrase	279	HTH-like domain, pfam13276; Integrase core domain, pfam00665, pfam13683; PHA02517, putative transposase OrfB	shn_30220 (26%)<br>AIH15778.1 (27%)
AWN88_01230	AMD58726.1	transposase	120	InsE, transposase and inactivated derivatives; HTH_Tnp_1, transposase, pfam01527	shn_30320 (32%)<br>AIH15784.1 (32%)
AWN88_01235	AMD56929.1	isocitrate lyase	115	TnpB_IS66, IS66 Orf2 like protein, pfam05717	shn_30315 (52%)<br>AIH15785.1 (53%)
AWN88_01240	AMD56930.1	transposase	521	LZ_Tnp_IS66, transposase C of IS166 homeodomain, pfam13007; zf-IS66, zinc-finger binding domain of transposase IS66, pfam13005; DDE_Tnp_IS66, transposase IS66 family, pfam03050; DDE_Tnp_IS66_C, IS66 C-terminal element, pfam13817	shn_30310 (39%)<br>AIH15786.1 (39%)
AWN88_01250	AMD56932.1	transposase	448	MULE, MULE transposase domain, cl24015	
AWN88_01265	AMD56935.1	transposase	109	HTH, helix-turn-helix domains, cl21459; InsE, transposase and inactivated derivatives	shn_30245 (83%, N-terminus), AIH15783.1 (83%)
AWN88_01270	AMD56936.1	transposase	102	HTH_21, HTH-like domain, pfam13276	shn_30245 (99%, C-terminus)<br>AIH15783.1 (100%)
AWN88_01275	AMD56937.1	group II intron reverse transcriptase/maturase	504	RT_G2_intron, reverse transcriptases (RTs) with group II intron origin; GIIM	AIH15789.1 (91%)
AWN88_01285	AMD58727.1	DDE endonuclease	346	HTH_29, winged helix-turn helix, pfam13551; rve, integrase core domain, cl21549	shn_30250 (99%)
AWN88_01400	AMD56957.1	transposase	121	DDE_3, DDE superfamily endonuclease, pfam13358	shn_35330 (41%, C-terminus)
AWN88_01405	AMD56958.1	transposase	127		shn_35330 (36%, N-terminus)

AWN88_01420	AMD56961.1	DNA invertase	305	PinE, site-specific DNA recombinase related to the DNA invertase Pin; SR_ResInv, Serine Recombinase (SR) family, Resolvase and Invertase subfamily; Recombinase, pfam07508	
AWN88_01430	AMD56963.1	transposase	307	P-loop_NTPase, P-loop containing Nucleoside Triphosphate Hydrolases,cl21455;	
AWN88_01450	AMD56966.1	integrase	382	INT_Cre_C, C-terminal catalytic domain of Cre recombinase (also called integrase), cd00799	shn_30405 (79%)
***Shinella* sp. HZN7**					
shn_35330	774..1720	IS630 family transposase		Frameshifted, pseudo	
shn_30215	WP_064334249.1	IS110 family transposase	223	Transposase_20 , PF02371	
shn_30220	19809..20968	IS3 family transposase		Frameshifted, pseudo	
shn_30245	25600..26798	IS3 family transposase		Frameshifted, pseudo	
shn_30250	WP_064334252.1	IS630 family transposase	358	DDE endonucease	AWN88_01285 (99%)
shn_30310	WP_064334257.1	transposase	551	LZ_Tnp_IS66, Transposase C of IS166 homeodomain; zf-IS66, zinc-finger binding domain of transposase; DDE_Tnp_IS66, Transposase IS66 family; DDE_Tnp_IS66_C, IS66 C-terminal element	AWN88_01240 (40%)<br>AIH15786.1 (97%)
shn_30315	WP_064334258.1	hypothetical protein	115	TnpB_IS66, IS66 Orf2 like protein	AWN88_01235 (52%)<br>AIH15785.1 (97%)
shn_30320	WP_037407864.1	transposase	136	HTH, helix-turn-helix domains, cl21459	AWN88_01230 (32%)<br>AIH15784.1 (78%)
shn_30370	WP_084438677.1	IS21 family transposase	511	Rve, Integrase core domain	
shn_30390	WP_064334265.1	transposase	553	DDE_Tnp_IS66, Transposase IS66 family	AIH15757.1 (63%, 158 amino acids)
shn_30405	WP_064334268.1	integrase	387	Phage_integrase, Phage integrase family	AWN88_00875 (75%)
shn_30425	WP_064332397.1	integrase	511	Rve, Integrase core domain	
***Ochrobactrum* sp. SJY1**					
229..2445	AIH15730.1	transposase	738	DDE_Tnp_Tn3, Tn3 transposase DDE domain, pfam01526	AWN88_00475 (99%)<br>AWN88_00400 (99%)
6187..7725	AIH15734.1	mobile element protein	512	InsE, transposase and inactivated derivatives; PHA02517, putative transposase OrfB; HTH_21, HTH-like domain, pfam13276; rve, integrase core domain, pfam00665; rve_2 , integrase core domain, pfam13333	shn_30245 (29%, partial)<br>shn_30220 (25%, partial)

28070..29203	AIH15755.1	mobile element protein	377	INT_Cre_C, C-terminal catalytic domain of Cre recombinase (also called integrase), cd00799	shn_30405 (45%, partial)
29471..29947	AIH15757.1	mobile element protein	158	rod shape-determining protein MreC, cl19252; Cupredoxin superfamily, cl19115	shn_30390 (50%, partial)
29995..32961	AIH15758.1	mobile element protein	988	Domain of unknown function (DUF4158), pfam13700; DDE_Tnp_Tn3, Tn3 transposase DDE domain, pfam01526	AWN88_00475 (41%)<br><br>AWN88_00400 (41%)
50419..50583	AIH15776.1	mobile element protein	54	DDE_Tnp_IS66_C, IS66 C-terminal element, pfam13817	shn_30310 (66%, partial)<br><br>AWN88_01240 (46%, partial)
50953..51783	AIH15778.1	mobile element protein	276	PHA02517, putative transposase OrfB; HTH_21, HTH-like domain, pfam13276; rve, integrase core domain, pfam00665; rve_3, integrase core domain, pfam13683	shn_30220-shn_30225 (83%)<br><br>shn_30245 (31%)<br><br>AWN88_01165 (28%)
51780..52091	AIH15779.1	fig01075454: hypothetical protein	103	InsE, transposase and inactivated derivatives; HTH, helix-turn-helix domains, cl21459	shn_30225 (97%)
54155..55351	AIH15782.1	mobile element protein	398	Transposase_mut, transposase, mutator family, pfam00872; MULE, MULE transposase domain, pfam10551	
55494..55823	AIH15783.1	mobile element protein	109	HTH, helix-turn-helix domains, cl21459; InsE, transposase and inactivated derivatives	AWN88_01265 (99%), shn_30240-30245 (84%)
55967..56377	AIH15784.1	transposase	136	InsE, transposase and inactivated derivatives; HTH, helix-turn-helix domains, cl21459	shn_30320 (78%), AWN88_01230 (28%)
56374..56721	AIH15785.1	mobile element protein	115	TnpB_IS66, IS66 Orf2 like protein, pfam05717	shn_30315-30320 (97%) , AWN88_01230-01235 (53%)
56767..58422	AIH15786.1	mobile element protein	551	LZ_Tnp_IS66, transposase C of IS166 homeodomain, pfam13007; zf-IS, zinc-finger binding domain of transposase IS66, pfam13005; DDE_Tnp_IS66, transposase IS66 family, pfam03050; DDE_Tnp_IS66_C, IS66 C-terminal element, pfam13817	shn_30310 (90%)<br><br>AWN88_01240 (40%)
58504..58812	AIH15787.1	transposase	102	HTH_21, HTH-like domain, pfam13276	AWN88_01270 100%), shn_30245 (99%)
59446..60957	AIH15789.1	retron-type RNA-directed DNA polymerase	503	group_II_RT_mat, group II intron reverse transcriptase/maturase, TIGR04416; RT_G2_intron, reverse transcriptases (RTs) with group II intron origin; GIIM, group II intron, maturase-specific domain, pfam08388	AWN88_01275 (94%)
61058..61279	AIH15790.1	transposase	73	Rve, integrase core domain, pfam00665	shn_30245 (92%)

					AWN88_01280 (78%)
61247..61426	AIH15791.1	IS3 family transposase	59	IS3 family transposase	shn_30245 (98%)<br><br>AWN88_07925 (39%)
61382..61516	AIH15792.1	mobile element protein	44	PHA02517, putative transposase OrfB	shn_30245 (93%)
84035..84601	AIH15815.1	mobile element protein	188	COG3415, transposase; HTH_23, homeodomain-like domain, pfam13384; HTH_32, homeodomain-like domain, pfam13565; HTH_33, winged helix-turn helix, pfam13592	
84747..85172	AIH15816.1	mobile element protein	141	Rve, integrase core domain, cl21549	AWN88_01130 (32%, partial)
85292..85666	AIH15817.1	mobile element protein	124	HTH, helix-turn-helix domains, cl21459; InsE, transposase and inactivated derivatives	shn_30225 (23%)
85663..86040	AIH15818.1	mobile element protein	125	HTH_28, helix-turn-helix domain, pfam13518; HTH_32, homeodomain-like domain, pfam13565	

Table S5. Differently expressed genes when *A. tumefaciens* S33 grows in nicotine medium compared to glucose-ammonium medium.

locus	fpkm_value_S33Glu	fpkm_value_S33Nic	log2(fold_change)	*p*_value	type	gene_function
NZ_CP014259.1:1121514-1121859	465.023	1553.11	1.73979	0.04765	up	hypothetical protein
NZ_CP014259.1:1123553-1124129	91.3148	504.75	2.46665	0.0061	up	photosystem reaction center subunit H
NZ_CP014259.1:1126343-1127333	185.065	660.118	1.83469	0.042	up	histidine kinase
NZ_CP014259.1:1128362-1129135	153.073	766.453	2.32398	0.0437	up	RNA polymerase sigma factor
NZ_CP014259.1:1129845-1133520	28.7823	7.26041	-1.98706	0.01535	down	trifunctional transcriptional regulator/proline dehydrogenase/L-glutamate gamma-semialdehyde dehydrogenase
NZ_CP014259.1:1134402-1135182	977.265	259.216	-1.9146	0.04195	down	sulfonate ABC transporter
NZ_CP014259.1:1136406-1137936	590.589	96.8432	-2.60843	0.01945	down	aldehyde dehydrogenase
NZ_CP014259.1:1148166-1149027	5.02181	34.7615	2.79121	0.0019	up	membrane protein
NZ_CP014259.1:1149034-1149445	5.40437	27.2825	2.33578	0.0138	up	TIGR02588 family protein
NZ_CP014259.1:1149464-1150691	6.96927	30.6732	2.1379	0.00535	up	FAD-dependent oxidoreductase
NZ_CP014259.1:1151934-1154271	115.708	499.17	2.10904	0.03915	up	glucose dehydrogenase
NZ_CP014259.1:1161147-1162035	68.0693	224.191	1.71965	0.03135	up	ABC transporter permease
NZ_CP014259.1:1162037-1162739	49.2056	258.665	2.39419	0.00335	up	ABC transporter ATP-binding protein

NZ_CP014259.1:1162740-1163526	144.33	801.711	2.47371	0.00905	up	ABC transporter ATP-binding protein
NZ_CP014259.1:1163555-1164764	124.734	1511.14	3.59871	0.0115	up	ABC transporter permease
NZ_CP014259.1:1189341-1191875	140.382	29.3109	-2.25984	0.04075	down	quinolinate synthetase
NZ_CP014259.1:1193579-1193768	518.51	1464.66	1.49812	0.04925	up	hypothetical protein
NZ_CP014259.1:1206087-1206801	142.761	24.7146	-2.53016	0.0021	down	aspartate racemase
NZ_CP014259.1:1206801-1207572	41.6617	10.2212	-2.02716	0.00615	down	class II glutamine amidotransferase
NZ_CP014259.1:1230163-1231129	423.911	119.099	-1.83161	0.0315	down	succinoglycan biosynthesis protein exov
NZ_CP014259.1:1231180-1232146	497.081	110.482	-2.16968	0.02665	down	succinoglycan biosynthesis protein exow
NZ_CP014259.1:1234113-1235262	289.209	66.1333	-2.12867	0.01595	down	succinoglycan biosynthesis protein exoh
NZ_CP014259.1:1235266-1236058	313.042	87.8451	-1.83332	0.0393	down	endo-1%2C3-1%2C4-beta-glycanase
NZ_CP014259.1:1254692-1254878	33.0014	270.927	3.0373	0.00575	up	hypothetical protein
NZ_CP014259.1:1255062-1256292	29.0048	169.38	2.5459	0.00605	up	glucose dehydrogenase
NZ_CP014259.1:1259662-1260679	37.8661	365.341	3.27027	0.00025	up	ABC transporter substrate-binding protein
NZ_CP014259.1:1264147-1265068	15.5129	43.1946	1.47738	0.04715	up	gluconolactonase
NZ_CP014259.1:1284651-1285533	1094.58	33.7403	-5.01977	5.00E-05	down	membrane protein
NZ_CP014259.1:12	4870.22	66.434	-6.19592	0.0068	down	ornithine cyclodeaminase

85556-1286612						
NZ_CP014259.1:1286625-1287546	3733.3	58.7217	-5.99041	5.00E-05	down	arginase
NZ_CP014259.1:1290655-1291642	34.1703	164.506	2.26732	0.00605	up	3-oxoacyl-ACP synthase
NZ_CP014259.1:1297227-1298933	1.73275	89.9089	5.69733	0.00025	up	copper resistance protein
NZ_CP014259.1:1298947-1300297	3.30639	98.4431	4.89596	5.00E-05	up	copper oxidase
NZ_CP014259.1:1300332-1300809	8.94384	342.405	5.25867	5.00E-05	up	copper oxidase
NZ_CP014259.1:1300832-1301117	19.2692	520.335	4.75507	5.00E-05	up	hypothetical protein
NZ_CP014259.1:1301203-1302040	6.0392	34.3856	2.50937	0.0029	up	membrane protein
NZ_CP014259.1:1305723-1306575	34.5981	103.817	1.58528	0.0452	up	LysR family transcriptional regulator
NZ_CP014259.1:1325849-1326701	22.3415	67.1233	1.58709	0.0301	up	ectoine/hydroxyectoine ABC transporter substrate-binding protein EhuB
NZ_CP014259.1:1403810-1404200	253.985	34.7831	-2.86829	0.001	down	acyl-CoA thioesterase
NZ_CP014259.1:1443493-1444564	20.59	60.4915	1.55479	0.0467	up	ABC transporter ATP-binding protein
NZ_CP014259.1:1469151-1471257	53.3672	271.784	2.34844	0.01335	up	ligand-gated channel protein
NZ_CP014259.1:1480958-1481354	69.731	285.352	2.03287	0.01045	up	deaminase
NZ_CP014259.1:1519278-1520256	24.0104	245.694	3.35513	0.0003	up	LacI family transcriptional regulator
NZ_CP014259.1:1520372-1523864	3.23115	27.4276	3.08551	0.03485	up	lipase

NZ_CP014259.1:1523865-1524714	7.18641	21.3622	1.57172	0.03015	up	tagatose 3-epimerase
NZ_CP014259.1:1525472-1526342	14.5155	44.6335	1.62053	0.0253	up	dolichol monophosphate mannose synthase
NZ_CP014259.1:1576371-1577751	191.522	29.9607	-2.67636	0.0027	down	hypothetical protein
NZ_CP014259.1:1623264-1623737	28.2605	8.32306	-1.7636	0.03245	down	TetR family transcriptional regulator
NZ_CP014259.1:1623864-1625667	261.98	34.9447	-2.90631	0.0034	down	FAD-dependent oxidoreductase
NZ_CP014259.1:1625785-1626859	731.159	51.1632	-3.83701	0.0003	down	zinc-binding dehydrogenase
NZ_CP014259.1:1634881-1635865	182.003	58.2486	-1.64367	0.04515	down	spermidine/putrescine ABC transporter permease
NZ_CP014259.1:1635874-1636666	197.772	55.1143	-1.84334	0.0197	down	spermidine/putrescine ABC transporter permease
NZ_CP014259.1:1636669-1637740	198.634	39.3002	-2.3375	0.00985	down	spermidine/putrescine ABC transporter ATP-binding protein
NZ_CP014259.1:1637793-1639275	573.659	96.4943	-2.57168	0.01795	down	FAD-dependent oxidoreductase
NZ_CP014259.1:1639285-1639879	518.393	93.8993	-2.46486	0.00545	down	2-hydroxychromene-2-carboxylate isomerase
NZ_CP014259.1:1639886-1640393	367.082	55.9005	-2.71517	0.00155	down	hypothetical protein
NZ_CP014259.1:1640394-1640973	284.014	59.3958	-2.25753	0.0055	down	alkylhydroperoxidase
NZ_CP014259.1:1640973-1642311	82.1267	23.8126	-1.78613	0.02725	down	MATE family efflux transporter
NZ_CP014259.1:1702417-1702792	31.2218	93.1684	1.57729	0.02935	up	hypothetical protein
NZ_CP014259.1:17	14.8605	136.527	3.19964	5.00E-05	up	sugar-binding protein

13457-1714726						
NZ_CP014259.1:1716003-1716471	19.6413	52.0532	1.4061	0.0465	up	AsnC family transcriptional regulator
NZ_CP014259.1:1718757-1719654	34.2944	240.404	2.80941	0.00085	up	LysR family transcriptional regulator
NZ_CP014259.1:1728783-1729818	8.06918	25.1267	1.63873	0.0237	up	oxidoreductase
NZ_CP014259.1:1757067-1758522	19.5857	71.4749	1.86764	0.01815	up	dihydropyrimidine dehydrogenase subunit A
NZ_CP014259.1:1802369-1803395	291.646	2357.83	3.01517	0.01935	up	fructose-bisphosphate aldolase
NZ_CP014259.1:1815311-1815815	42.9951	120.595	1.48792	0.04165	up	general stress protein
NZ_CP014259.1:1838003-1839077	119.764	497.484	2.05446	0.0284	up	membrane protein
NZ_CP014259.1:1839157-1839337	6.06032	0	#NAME?	0.02045	down	hypothetical protein
NZ_CP014259.1:1910119-1911352	52.4561	6.71755	-2.9651	0.0005	down	transcriptional regulator
NZ_CP014259.1:1912713-1914027	1195.26	93.7058	-3.67304	0.0006	down	sugar ABC transporter permease
NZ_CP014259.1:1914045-1914828	1289.91	85.2667	-3.91914	0.0009	down	sugar ABC transporter ATP-binding protein
NZ_CP014259.1:1922966-1923881	28.3119	4.48348	-2.65872	0.00295	down	myo-inositol catabolism protein
NZ_CP014259.1:1988336-1989869	14.2189	54.2396	1.93154	0.01575	up	D-ribose transporter ATP-binding protein
NZ_CP014259.1:1990085-1991096	67.4092	585.956	3.11977	0.00225	up	rhamnose ABC transporter substrate-binding protein
NZ_CP014259.1:1991146-1991977	28.5303	137.137	2.26506	0.00505	up	DeoR family transcriptional regulator

NZ_CP014259.1:1992174-1994271	13.8605	163.405	3.5594	0.0003	up	short-chain dehydrogenase
NZ_CP014259.1:1994460-1995753	9.06399	63.5726	2.81019	0.0012	up	sugar isomerase
NZ_CP014259.1:2025686-2026835	8.46901	24.0072	1.5032	0.0432	up	mandelate racemase
NZ_CP014259.1:2027257-2028286	117.672	33.4634	-1.81412	0.02195	down	oxidoreductase
NZ_CP014259.1:2051436-2052507	39.7626	5.77431	-2.78369	0.001	down	alkanesulfonate monooxygenase
NZ_CP014259.1:2052903-2053506	623.494	149.751	-2.05781	0.0232	down	esterase
NZ_CP014259.1:2053694-2053892	708.958	219.328	-1.69261	0.02445	down	hypothetical protein
NZ_CP014259.1:2055410-2055956	1066.8	188.37	-2.50165	0.0122	down	flavin reductase
NZ_CP014259.1:2056004-2056949	1192.13	208.993	-2.51201	0.0163	down	acyl-CoA dehydrogenase
NZ_CP014259.1:2060179-2061367	54.1051	17.3232	-1.64305	0.0406	down	amidohydrolase
NZ_CP014259.1:2061388-2061943	75.9903	26.3956	-1.52552	0.03685	down	acetyltransferase
NZ_CP014259.1:2061989-2062868	53.5041	15.6132	-1.77688	0.0168	down	hypothetical protein
NZ_CP014259.1:2063018-2063762	73.119	20.2762	-1.85046	0.01655	down	arginine ABC transporter ATP-binding protein
NZ_CP014259.1:212051-212246	0	13.5046	inf	0.02245	up	hypothetical protein
NZ_CP014259.1:2129959-2130520	281.282	33.3845	-3.07476	0.00025	down	FMN reductase
NZ_CP014259.1:21	221.024	27.7235	-2.99503	0.0011	down	monooxygenase

30571-2131753						
NZ_CP014259.1:2131866-2132970	679.375	76.1953	-3.15643	0.00125	down	alkanesulfonate monooxygenase
NZ_CP014259.1:2133077-2137757	232.044	20.9133	-3.47191	0.0421	down	ABC transporter permease
NZ_CP014259.1:2137795-2139724	421.254	29.1169	-3.85476	0.00195	down	peptide ABC transporter substrate-binding protein
NZ_CP014259.1:2139901-2142190	52.2926	10.0566	-2.37847	0.00365	down	pseudo=true
NZ_CP014259.1:2142228-2143605	121.638	20.5813	-2.5632	0.0017	down	2-hydroxy-acid oxidase
NZ_CP014259.1:2143701-2144304	364.079	60.7029	-2.58442	0.0055	down	2-hydroxychromene-2-carboxylate isomerase
NZ_CP014259.1:2144316-2144889	168.788	37.6837	-2.1632	0.0077	down	transcriptional regulator
NZ_CP014259.1:2144940-2145969	108.71	20.9689	-2.37415	0.003	down	2-dehydropantoate 2-reductase
NZ_CP014259.1:2146270-2147113	161.821	45.3716	-1.83454	0.0207	down	NADPH-dependent oxidoreductase
NZ_CP014259.1:2182931-2184119	1692.79	74.3252	-4.50941	0.0041	down	acyl-CoA dehydrogenase
NZ_CP014259.1:2207403-2208798	19.0201	6.00358	-1.66363	0.0282	down	amidase
NZ_CP014259.1:2218507-2219821	107.464	936.424	3.12331	0.00235	up	sugar ABC transporter substrate-binding protein
NZ_CP014259.1:2219987-2220860	27.6457	134.716	2.28479	0.0043	up	sugar ABC transporter permease
NZ_CP014259.1:2220872-2221697	22.8329	104.398	2.19291	0.0065	up	mannitol ABC transporter permease
NZ_CP014259.1:2221709-2222708	18.0201	106.346	2.5611	0.0024	up	sugar ABC transporter ATP-binding protein

NZ_CP014259.1:2222827-2224312	21.1702	96.1587	2.18338	0.0068	up	mannitol dehydrogenase
NZ_CP014259.1:2231070-2232045	32.0575	266.099	3.05323	0.0012	up	LacI family transcriptional regulator
NZ_CP014259.1:2232250-2233216	501.716	2742.8	2.45071	0.04605	up	transaldolase
NZ_CP014259.1:2249181-2250765	31.0171	8.81687	-1.81473	0.01685	down	MFS transporter
NZ_CP014259.1:2257677-2258307	30.9404	96.5157	1.64127	0.0289	up	nitroreductase
NZ_CP014259.1:231487-231775	1044.57	282.871	-1.88469	0.01795	down	conjugal transfer protein TraC
NZ_CP014259.1:2294016-2295189	83.082	366.233	2.14015	0.014	up	amino acid-binding protein
NZ_CP014259.1:2307971-2308733	36.0673	107.292	1.57278	0.0466	up	glutamine ABC transporter ATP-binding protein
NZ_CP014259.1:2309628-2310279	9.61466	32.7134	1.76657	0.01685	up	amino acid ABC transporter permease
NZ_CP014259.1:2310291-2310960	8.83811	52.2656	2.56405	0.00245	up	ABC transporter permease
NZ_CP014259.1:2314243-2315041	15.0536	73.2974	2.28365	0.0024	up	3-oxoadipate enol-lactonase
NZ_CP014259.1:2357755-2359075	215.127	873.77	2.02206	0.04905	up	glycerol-3-phosphate ABC transporter substrate-binding protein
NZ_CP014259.1:2411245-2412388	33.5843	149.651	2.15574	0.00915	up	oxidoreductase
NZ_CP014259.1:2431674-2434267	107.341	21.9858	-2.28756	0.01865	down	aliphatic sulfonate ABC transporter
NZ_CP014259.1:2434418-2435744	188.597	46.8837	-2.00815	0.02675	down	nitrilotriacetate monooxygenase component A
NZ_CP014259.1:24	5.23087	20.1064	1.94253	0.01205	up	chemotaxis protein

42138-2443920						
NZ_CP014259.1:2445148-2445562	11.3226	41.175	1.86257	0.0164	up	hypothetical protein
NZ_CP014259.1:252633-254067	8.93411	50.3447	2.49444	0.0036	up	NAD(P) transhydrogenase subunit beta
NZ_CP014259.1:2458290-2459448	20.4598	6.88657	-1.57093	0.03575	down	class C beta-lactamase
NZ_CP014259.1:254095-255679	8.3343	87.8434	3.3978	0.0001	up	NAD(P) transhydrogenase subunit alpha
NZ_CP014259.1:256400-256583	0	107.324	inf	5.00E-05	up	hypothetical protein
NZ_CP014259.1:258378-258699	8.69004	117.036	3.75144	0.00415	up	pseudo=true
NZ_CP014259.1:258727-259337	14.9168	1922.06	7.00957	5.00E-05	up	pseudo=true
NZ_CP014260.1:18518-21023	9.67451	135.284	3.80566	0.0001	up	ATPase
NZ_CP014260.1:21034-21370	8.43728	96.008	3.5083	0.00395	up	hypothetical protein
NZ_CP014260.1:21381-21870	11.3152	112.067	3.30802	0.00075	up	Cu(I)-responsive transcriptional regulator
NZ_CP014260.1:21937-23771	13.9556	112.334	3.00888	0.00865	up	ABC transporter permease
NZ_CP014259.1:259746-260769	30.737	182.104	2.56671	0.0016	up	hypothetical protein
NZ_CP014260.1:21937-23771	12.3509	98.4439	2.99469	0.0107	up	ABC transporter permease
NZ_CP014260.1:24989-26000	19.903	62.3859	1.64824	0.0227	up	nickel ABC transporter ATP-binding protein
NZ_CP014260.1:28115-28370	59.8195	1701.24	4.82982	5.00E-05	up	copper chaperone

NZ_CP014260.1:45478-45853	192.529	63.3247	-1.60424	0.0294	down	TIGR02594 family protein
NZ_CP014260.1:45902-46142	54.0459	219.503	2.02198	0.01	up	hypothetical protein
NZ_CP014260.1:50390-50651	80.057	27.5218	-1.54045	0.0465	down	hypothetical protein
NZ_CP014259.1:264280-265945	16.5721	331.022	4.3201	5.00E-05	up	electron transfer flavoprotein-ubiquinone oxidoreductase
NZ_CP014259.1:266056-266407	11.3119	485.983	5.42499	0.0002	up	hypothetical protein
NZ_CP014259.1:266505-267435	44.7696	3518.5	6.2963	0.00015	up	electron transfer flavoprotein subunit beta
NZ_CP014259.1:267512-268262	47.2122	3916.53	6.37427	0.0002	up	electron transfer flavoprotein subunit beta
NZ_CP014259.1:268327-268540	243.228	9641.61	5.30889	5.00E-05	up	hypothetical protein
NZ_CP014259.1:268561-269914	90.4225	13708.9	7.24422	0.0328	up	MFS transporter
NZ_CP014259.1:270083-271537	41.9982	6642.84	7.30533	0.00205	up	para-nitrophenol 4-monooxygenase
NZ_CP014259.1:271599-272460	45.6245	3827.51	6.39045	0.00955	up	hypothetical protein
NZ_CP014259.1:272582-272960	136.334	6502.5	5.57577	0.00095	up	hypothetical protein
NZ_CP014260.1:189261-190113	230.069	44.5083	-2.36992	0.0037	down	hypothetical protein
NZ_CP014259.1:273062-275078	121.913	2627.11	4.42956	0.02195	up	hypothetical protein
NZ_CP014259.1:275272-275680	12.2644	139.284	3.50547	0.0003	up	hypothetical protein
NZ_CP014260.1:22	48.6267	3.83867	-3.66307	0.00095	down	multidrug DMT transporter permease

7838-228768						
NZ_CP014259.1:278641-279988	2.80361	10.4309	1.8955	0.0142	up	transposase
NZ_CP014259.1:280263-282474	48.4621	506.969	3.38697	0.00125	up	chemotaxis protein
NZ_CP014259.1:283368-284403	122.901	7038.91	5.83978	0.00555	up	putrescine/spermidine ABC transporter substrate-binding protein
NZ_CP014260.1:276728-277067	33.582	100.852	1.58649	0.0319	up	hypothetical protein
NZ_CP014260.1:300673-301813	1319.49	113.162	-3.54352	0.0176	down	glycine cleavage system aminomethyltransferase T
NZ_CP014260.1:306870-307374	7.23585	23.5638	1.70333	0.03105	up	hypothetical protein
NZ_CP014259.1:291089-292124	141.015	5902.87	5.3875	0.0128	up	putrescine/spermidine ABC transporter substrate-binding protein
NZ_CP014259.1:293856-294930	43.2709	3875.89	6.48499	0.00825	up	spermidine/putrescine ABC transporter ATP-binding protein
NZ_CP014259.1:295106-295739	28.9885	4835.76	7.38212	5.00E-05	up	carbamoylsarcosine amidase
NZ_CP014259.1:295800-296829	24.9847	12309.7	8.94454	0.03205	up	leucyl aminopeptidase
NZ_CP014260.1:407901-408339	117.117	43.7968	-1.41906	0.0489	down	membrane protein
NZ_CP014259.1:296854-297637	13.9544	5307.77	8.57124	0.00245	up	alpha/beta hydrolase
NZ_CP014259.1:297649-298399	18.2106	8084.81	8.79429	0.00095	up	Asp/Glu racemase
NZ_CP014260.1:426607-426895	6.24468	0	#NAME?	0.01285	down	hypothetical protein
NZ_CP014259.1:298937-299345	39.9672	7550.02	7.56152	0.00015	up	hypothetical protein

NZ_CP014259.1:299628-301026	74.8364	9053.21	6.91855	0.03385	up	aldehyde dehydrogenase
NZ_CP014259.1:301055-302369	178.319	10441.2	5.87168	0.0317	up	hypothetical protein
NZ_CP014259.1:302475-302907	167.75	5045.96	4.91074	0.00275	up	pseudoazurin
NZ_CP014259.1:302988-305702	21.9143	3044.96	7.11841	0.00045	up	hypothetical protein
NZ_CP014259.1:305978-306653	21.1065	354.632	4.07056	0.00015	up	TetR family transcriptional regulator
NZ_CP014260.1:502392-503718	1169.83	65.9277	-4.14927	0.0096	down	manganese transporter
NZ_CP014260.1:528770-529691	169.727	56.5899	-1.5846	0.04995	down	methylenetetrahydrofolate reductase
NZ_CP014259.1:311238-312467	42.8238	169.13	1.98165	0.03165	up	hypothetical protein
NZ_CP014260.1:569533-569803	30.3857	137.316	2.17604	0.0064	up	hypothetical protein
NZ_CP014260.1:672653-672893	123.601	21.5513	-2.51984	0.01055	down	hypothetical protein
NZ_CP014260.1:690099-690366	1545.29	379.685	-2.025	0.01195	down	Usg protein%2C probable subunit of phosphoribosylanthranilate isomerase
NZ_CP014260.1:691517-692483	344.82	62.5953	-2.46172	0.00715	down	aliphatic sulfonate ABC transporter substrate-binding protein
NZ_CP014260.1:710979-711981	11.0829	33.7962	1.60852	0.0286	up	serine hydrolase
NZ_CP014260.1:781706-782726	260.321	1724.89	2.72814	0.03965	up	ketol-acid reductoisomerase
NZ_CP014260.1:784923-785970	163.577	41.3313	-1.98466	0.0171	down	hydroxyacid dehydrogenase
NZ_CP014260.1:78	143.408	31.4871	-2.1873	0.0081	down	NADH dehydrogenase

6039-787305						
NZ_CP014260.1:795019-795592	97.6146	633.822	2.69891	0.00305	up	acetolactate synthase small subunit
NZ_CP014260.1:795611-797405	65.9401	656.276	3.31508	0.00265	up	acetolactate synthase large subunit
NZ_CP014260.1:844713-845673	1177.04	226.207	-2.37945	0.02165	down	UDP-3-O-[3-hydroxymyristoyl] N-acetylglucosamine deacetylase
NZ_CP014260.1:851505-852810	50.9594	404.633	2.98919	0.0008	up	MFS transporter
NZ_CP014260.1:870241-870781	38.8072	11.5464	-1.74888	0.01815	down	lytic transglycosylase
NZ_CP014260.1:886198-889065	695.483	24.9227	-4.80248	0.0086	down	CoA-transferase
NZ_CP014260.1:886198-889065	701.301	31.8807	-4.45928	0.0249	down	thiamine pyrophosphate-binding protein
NZ_CP014260.1:964994-965594	18.7383	79.1051	2.07778	0.00715	up	hypothetical protein
NZ_CP014260.1:1029031-1030741	101.287	1348.75	3.7351	0.0056	up	2-isopropylmalate synthase
NZ_CP014260.1:1088414-1088758	16.0215	313.687	4.29124	5.00E-05	up	quaternary ammonium transporter
NZ_CP014260.1:1088760-1089399	20.8718	392.784	4.23411	0.0001	up	TetR family transcriptional regulator
NZ_CP014260.1:1146527-1147724	290.639	88.5932	-1.71396	0.04345	down	hemolysin secretion protein D
NZ_CP014259.1:377267-378476	18.062	55.51	1.61979	0.0471	up	ABC transporter permease
NZ_CP014260.1:1171172-1171943	7.64309	25.6639	1.74751	0.0174	up	gluconate 5-dehydrogenase
NZ_CP014260.1:1198785-1199688	116.765	427.319	1.87171	0.0291	up	RNA polymerase factor sigma-32

NZ_CP014260.1:1236197-1237388	23.6879	113.279	2.25765	0.0058	up	MexE family multidrug efflux RND transporter periplasmic adaptor subunit
NZ_CP014260.1:1237521-1240716	14.453	56.8905	1.97682	0.0156	up	multidrug efflux RND transporter permease subunit
NZ_CP014260.1:1243843-1244718	106.372	25.8632	-2.04014	0.0105	down	pyridoxamine kinase
NZ_CP014260.1:1248293-1250177	65.8005	1147.51	4.12427	0.003	up	MFS transporter
NZ_CP014260.1:1259228-1260113	24.7626	1020.13	5.36444	0.00015	up	dihydrodipicolinate synthase family protein
NZ_CP014260.1:1260133-1260790	46.19	502.347	3.44303	0.00065	up	GntR family transcriptional regulator
NZ_CP014260.1:1260890-1263993	92.0385	1258.13	3.7729	0.0453	up	ABC transporter substrate-binding protein
NZ_CP014260.1:1264025-1265084	59.0166	248.136	2.07194	0.0228	up	sugar ABC transporter ATP-binding protein
NZ_CP014260.1:1293461-1295339	45.3168	912.958	4.33243	5.00E-05	up	membrane protein
NZ_CP014260.1:1295404-1296415	156.369	2040.35	3.70579	0.00865	up	ABC transporter substrate-binding protein
NZ_CP014260.1:1296478-1297294	42.3481	312.858	2.88514	0.00145	up	3-oxoacyl-ACP reductase
NZ_CP014260.1:1302698-1303659	123.535	23.7365	-2.37974	0.0204	down	DNA-binding protein
NZ_CP014260.1:1329598-1330882	55.2571	11.8828	-2.21729	0.0067	down	FAD-dependent oxidoreductase
NZ_CP014260.1:1375581-1376067	49.4838	161.502	1.70652	0.0341	up	hypothetical protein
NZ_CP014260.1:1380537-1381719	110.66	565.326	2.35294	0.01465	up	oxidoreductase
NZ_CP014260.1:13	45.6308	169.037	1.88926	0.0184	up	transcriptional regulator

82159-1382912						
NZ_CP014260.1:14 88555-1489965	67.3529	256.95	1.93167	0.02435	up	isopropylmalate isomerase
NZ_CP014260.1:14 98582-1499509	427.918	58.9104	-2.86074	0.00135	down	homoserine O-succinyltransferase
NZ_CP014260.1:15 51929-1552280	21.005	165.879	2.98133	0.0006	up	nitrogen regulatory protein P-II 1
NZ_CP014260.1:15 52309-1553656	29.152	136.654	2.22886	0.00615	up	ammonia channel protein
NZ_CP014260.1:16 16779-1618345	7.42329	31.3075	2.07638	0.0061	up	D-ribose transporter ATP-binding protein
NZ_CP014260.1:16 73776-1675387	53.4569	1821.57	5.09066	0.00035	up	phosphoenolpyruvate carboxykinase (ATP)
NZ_CP014259.1:41 7363-417576	3.15072	34.7799	3.4645	0.0422	up	hypothetical protein
NZ_CP014260.1:16 86789-1687503	331.626	104.567	-1.66512	0.0444	down	hypothetical protein
NZ_CP014260.1:17 02761-1703262	9.92432	47.7151	2.26541	0.00535	up	hypothetical protein
NZ_CP014260.1:17 03287-1704316	14.8862	163.606	3.45818	5.00E-05	up	5-methyltetrahydropteroyltriglutamate--homocysteine methyltransferase
NZ_CP014260.1:17 04353-1705325	30.5408	134.046	2.13392	0.0083	up	pseudo=true
NZ_CP014260.1:17 05950-1706787	20.0363	5.22261	-1.93977	0.01345	down	acyl-CoA thioesterase
NZ_CP014260.1:17 08242-1708464	1401	52.9556	-4.72553	5.00E-05	down	NrdH-redoxin
NZ_CP014260.1:17 08476-1711025	586.41	32.2472	-4.18466	0.0044	down	ribonucleotide-diphosphate reductase subunit alpha
NZ_CP014260.1:17 11042-1712032	549.466	46.5961	-3.55975	0.00025	down	ribonucleotide-diphosphate reductase subunit beta

NZ_CP014260.1:1712300-1713410	77.0438	14.6293	-2.39682	0.00325	down	transposase
NZ_CP014259.1:421463-421694	17.1805	64.0443	1.8983	0.0479	up	hypothetical protein
NZ_CP014260.1:1728772-1728874	3789.02	0	#NAME?	0.02045	down	pseudo=true
NZ_CP014260.1:1793346-1793574	10.8491	0	#NAME?	0.01285	down	hypothetical protein
NZ_CP014260.1:1813060-1814293	32.0068	148.123	2.21035	0.00755	up	ROK family transcriptional regulator
NZ_CP014260.1:1814557-1815580	36.3008	3689.29	6.6672	0.0088	up	sugar ABC transporter
NZ_CP014260.1:1815803-1818294	16.1808	910.728	5.81467	0.0005	up	ABC transporter permease
NZ_CP014260.1:1815803-1818294	11.1146	486.102	5.45073	0.00725	up	sugar ABC transporter ATP-binding protein
NZ_CP014260.1:1818946-1819168	2373.64	89.0263	-4.73673	5.00E-05	down	NrdH-redoxin
NZ_CP014260.1:1819179-1819578	1243.03	68.3111	-4.1856	5.00E-05	down	ribonucleotide reductase assembly protein NrdI
NZ_CP014260.1:1819670-1821752	1243.85	72.8791	-4.09317	0.0019	down	ribonucleotide-diphosphate reductase subunit alpha
NZ_CP014260.1:1821768-1822743	843.332	78.4572	-3.42612	0.00355	down	ribonucleotide-diphosphate reductase subunit beta
NZ_CP014260.1:1868026-1869010	136.558	733.358	2.425	0.01015	up	hypothetical protein
NZ_CP014260.1:1869109-1869571	94.6664	333.015	1.81467	0.02005	up	membrane protein
NZ_CP014259.1:439621-439825	340.199	1536.96	2.17563	0.0073	up	hypothetical protein
NZ_CP014260.1:19	175.568	644.549	1.87626	0.04115	up	ABC transporter permease

50128-1951040						
NZ_CP014260.1:1975675-1976125	91.9373	750.732	3.02958	0.0016	up	hypothetical protein
NZ_CP014260.1:1981011-1981329	153.351	1053.01	2.77961	0.00295	up	hypothetical protein
NZ_CP014260.1:2012170-2012878	24.1756	96.0539	1.99029	0.01575	up	GntR family transcriptional regulator
NZ_CP014260.1:2012982-2013483	100.574	1217.38	3.59746	0.0006	up	transcriptional regulator
NZ_CP014260.1:2013564-2014428	83.4295	2320.55	4.79776	0.00265	up	ABC transporter permease
NZ_CP014260.1:2136737-2137775	106.834	13.4161	-2.99333	0.00045	down	proline racemase
NZ_CP014260.1:2137887-2138925	64.4131	17.4157	-1.88697	0.0195	down	dihydroorotase
NZ_CP014259.1:464721-466374	15.5323	102.968	2.72886	0.00095	up	MFS transporter
NZ_CP014259.1:466489-466834	21.7008	124.78	2.52357	0.0026	up	NAD-dependent formate dehydrogenase subunit delta
NZ_CP014260.1:2166021-2166984	214.013	773.525	1.85375	0.0385	up	2-nitropropane dioxygenase
NZ_CP014259.1:466905-469788	11.8548	112.082	3.24101	0.0004	up	formate dehydrogenase
NZ_CP014260.1:2182803-2183988	816.609	118.981	-2.77891	0.01355	down	O-succinylhomoserine sulfhydrylase
NZ_CP014259.1:469792-471825	10.8871	170.229	3.96679	0.00125	up	formate dehydrogenase
NZ_CP014259.1:469792-471825	16.1796	420.545	4.70002	0.04055	up	formate dehydrogenase
NZ_CP014260.1:2198728-2199340	338.074	32.6028	-3.37427	0.0002	down	hypothetical protein

NZ_CP014260.1:2199411-2200053	97.3488	20.1951	-2.26916	0.00455	down	hypothetical protein
NZ_CP014260.1:2216863-2217295	15.5642	51.9644	1.73929	0.0228	up	hypothetical protein
NZ_CP014260.1:2296719-2297745	13.3899	61.7855	2.20612	0.00865	up	transcriptional regulator
NZ_CP014260.1:2298019-2299381	37.8911	677.806	4.16094	0.00045	up	alpha-glucosides-binding periplasmic protein AglE
NZ_CP014260.1:2299486-2300500	18.2949	229.09	3.6464	5.00E-05	up	alpha-glucoside ABC transporter permease
NZ_CP014260.1:2300501-2301662	15.9977	143.378	3.16389	0.0002	up	alpha-glucoside ABC transporter permease
NZ_CP014260.1:2301699-2303355	20.4074	160.629	2.97657	0.00125	up	alpha-glucosidase
NZ_CP014260.1:2303375-2304464	28.3897	164.674	2.53618	0.0023	up	sugar ABC transporter ATP-binding protein
NZ_CP014260.1:2306661-2308482	268.522	43.2616	-2.63388	0.00995	down	phosphogluconate dehydratase
NZ_CP014260.1:2407446-2407761	116.258	41.3602	-1.49101	0.0376	down	transcriptional regulator
NZ_CP014259.1:495297-496326	53.0183	8.47609	-2.64502	0.0008	down	trans-3-hydroxy-L-proline dehydratase
NZ_CP014260.1:2422916-2423198	454.808	101.995	-2.15677	0.0063	down	hypothetical protein
NZ_CP014259.1:496336-498031	18.4848	5.26149	-1.81279	0.0156	down	hypothetical protein
NZ_CP014260.1:2440958-2442002	102.015	17.1144	-2.5755	0.0016	down	membrane protein
NZ_CP014260.1:2443147-2443522	56.7442	504.495	3.15229	0.00015	up	hypothetical protein
NZ_CP014259.1:49	56.948	5.78499	-3.29926	0.0232	down	D-amino-acid dehydrogenase

8143-500753						
NZ_CP014259.1:498143-500753	104.244	9.66914	-3.43043	0.0211	down	amino acid ABC transporter ATP-binding protein
NZ_CP014260.1:2451117-2452752	354.473	4660.65	3.71679	0.02445	up	molecular chaperone GroEL
NZ_CP014260.1:2452825-2453122	254.466	2774.99	3.44694	0.00075	up	molecular chaperone GroES
NZ_CP014259.1:500765-501431	96.8638	12.2895	-2.97853	0.00015	down	ABC transporter permease
NZ_CP014259.1:501500-502307	368.348	41.2528	-3.1585	0.00075	down	amino acid ABC transporter substrate-binding protein
NZ_CP014259.1:502750-503788	118.428	25.6162	-2.20889	0.0069	down	oxidoreductase
NZ_CP014260.1:2501507-2502752	16.4772	5.25544	-1.64859	0.0247	down	(2Fe-2S)-binding protein
NZ_CP014260.1:2504012-2504477	85.0992	463.685	2.44593	0.0022	up	hypothetical protein
NZ_CP014259.1:504077-505043	196.792	66.1223	-1.57346	0.0452	down	dihydrodipicolinate synthase family protein
NZ_CP014260.1:2508280-2509039	192.921	687.552	1.83346	0.0305	up	enoyl-CoA hydratase
NZ_CP014260.1:2550248-2550440	127.318	357.536	1.48965	0.04255	up	hypothetical protein
NZ_CP014260.1:2559206-2559896	160.095	30.0268	-2.4146	0.003	down	haloacid dehalogenase
NZ_CP014260.1:2560831-2562353	10.9571	60.6755	2.46925	0.03595	up	hypothetical protein
NZ_CP014260.1:2627548-2628271	46.0073	16.3258	-1.49471	0.0422	down	SDR family oxidoreductase
NZ_CP014260.1:2639003-2639885	382.937	82.3326	-2.21757	0.0146	down	aminotransferase

NZ_CP014260.1:2649895-2650078	2233.02	200.917	-3.47433	5.00E-05	down	cobalt transporter subunit CbtB
NZ_CP014260.1:2650088-2650802	1327.11	120.862	-3.45686	0.00505	down	cobalt transporter
NZ_CP014260.1:2650805-2651120	393.216	57.3581	-2.77725	0.00135	down	hypothetical protein
NZ_CP014260.1:2666201-2667362	30.3874	124.51	2.03472	0.01165	up	AI-2E family transporter
NZ_CP014260.1:2697904-2700909	21.9647	618.208	4.81483	0.0026	up	ATPase
NZ_CP014260.1:2700955-2701156	90.993	1000.18	3.45835	0.0001	up	heavy metal transporter
NZ_CP014260.1:2701204-2701498	71.733	719.771	3.32683	5.00E-05	up	hypothetical protein
NZ_CP014260.1:2701542-2701791	61.7613	212.739	1.78431	0.0214	up	hypothetical protein
NZ_CP014260.1:2707114-2708215	1029.94	12.6767	-6.34423	5.00E-05	down	saccharopine dehydrogenase
NZ_CP014260.1:2753462-2753654	1274.68	61.8614	-4.36496	5.00E-05	down	hypothetical protein
NZ_CP014259.1:536945-538460	93.7369	8.11283	-3.53034	0.00025	down	choline-sulfatase
NZ_CP014259.1:538474-539428	359.096	15.493	-4.53468	5.00E-05	down	glycine/betaine ABC transporter substrate-binding protein
NZ_CP014260.1:2774447-2775044	27.7385	113.271	2.02982	0.0116	up	hypothetical protein
NZ_CP014260.1:2775203-2775542	287.32	2191.79	2.93138	0.0031	up	hypothetical protein
NZ_CP014260.1:2780950-2781292	267.096	1121.51	2.07001	0.0233	up	hypothetical protein
NZ_CP014260.1:27	6173.5	218.167	-4.82258	5.00E-05	down	hypothetical protein

95142-2795328						
NZ_CP014260.1:2831227-2832136	178.024	37.6431	-2.24162	0.0062	down	permease
NZ_CP014259.1:53637-53850	15.7639	132.809	3.07466	0.01965	up	hypothetical protein
NZ_CP014260.1:2909631-2909949	133.949	45.9418	-1.5438	0.0359	down	hypothetical protein
NZ_CP014259.1:54124-54718	38.0345	533.912	3.81122	0.0003	up	adenylate kinase
NZ_CP014259.1:54759-55389	74.2306	2452.52	5.04611	5.00E-05	up	3%2C4-dihydroxy-2-butanone-4-phosphate synthase
NZ_CP014259.1:571386-572280	13.6606	39.5126	1.5323	0.0385	up	oxidoreductase
NZ_CP014259.1:586444-587716	95.7084	366.875	1.93857	0.0424	up	sugar ABC transporter substrate-binding protein
NZ_CP014259.1:587765-589367	63.6825	205.156	1.68775	0.04605	up	FAD-dependent oxidoreductase
NZ_CP014259.1:589372-590443	36.2617	139.084	1.93943	0.01465	up	LacI family transcriptional regulator
NZ_CP014259.1:55733-57114	83.9328	4587.89	5.77245	0.0085	up	hypothetical protein
NZ_CP014259.1:57136-58998	55.4385	1970.96	5.15187	0.0224	up	hypothetical protein
NZ_CP014259.1:59065-60211	34.1692	734.784	4.42655	0.0008	up	thiolase
NZ_CP014259.1:60234-61380	118.566	2578.79	4.44293	0.04475	up	branched-chain amino acid ABC transporter substrate-binding protein
NZ_CP014259.1:61400-64944	68.2921	868.599	3.6689	0.0296	up	hypothetical protein
NZ_CP014259.1:669542-670760	23.5383	85.6797	1.86395	0.01965	up	arabinose efflux permease

NZ_CP014259.1:679633-680935	75.9498	259.105	1.77042	0.04165	up	ABC transporter substrate-binding protein
NZ_CP014259.1:64953-66366	48.9804	387.494	2.9839	0.00255	up	hypothetical protein
NZ_CP014259.1:692264-692453	4.55937	0	#NAME?	0.02045	down	hypothetical protein
NZ_CP014259.1:693508-694225	15.34	161.948	3.40017	0.00015	up	GntR family transcriptional regulator
NZ_CP014259.1:694287-695262	182.309	1040.1	2.51227	0.0154	up	ABC transporter substrate-binding protein
NZ_CP014259.1:66382-67021	79.9772	242.096	1.59792	0.04645	up	hypothetical protein
NZ_CP014259.1:695322-695895	123.493	485.377	1.97468	0.01225	up	ABC transporter permease
NZ_CP014259.1:744931-746098	482.876	91.9784	-2.39228	0.01355	down	alanine racemase
NZ_CP014259.1:752393-753740	16.1641	418.702	4.69505	5.00E-05	up	C4-dicarboxylate transporter
NZ_CP014259.1:785075-786835	161.75	33.9507	-2.25226	0.0123	down	exopolysaccharide biosynthesis protein
NZ_CP014259.1:786842-788099	409.499	58.0756	-2.81785	0.0043	down	sugar ABC transporter substrate-binding protein
NZ_CP014259.1:788283-788964	3248.25	342.552	-3.24527	0.0171	down	exopolysaccharide biosynthesis protein
NZ_CP014259.1:803926-805192	230.577	63.2553	-1.86599	0.03275	down	ABC transporter substrate-binding protein
NZ_CP014259.1:805255-807060	92.06	21.1069	-2.12486	0.0379	down	sugar ABC transporter permease
NZ_CP014259.1:807070-808099	90.5633	12.1039	-2.90346	0.0012	down	sugar ABC transporter ATP-binding protein
NZ_CP014259.1:80	247.74	27.3174	-3.18094	5.00E-05	down	glutamine amidotransferase

8154-808940						
NZ_CP014259.1:808958-810008	219.836	40.9945	-2.42292	0.0079	down	oxidoreductase
NZ_CP014259.1:844050-845592	28.5347	98.2612	1.7839	0.02765	up	D-ribose transporter ATP-binding protein
NZ_CP014259.1:845829-846759	121.275	557.33	2.20024	0.02165	up	rhizopine-binding protein
NZ_CP014259.1:81820-82336	69.9992	1660.13	4.56782	5.00E-05	up	hypothetical protein
NZ_CP014259.1:855839-856784	26.3731	5.1571	-2.35443	0.0046	down	isoaspartyl peptidase
NZ_CP014259.1:856828-859440	27.8115	2.14266	-3.69821	0.00515	down	hydantoinase
NZ_CP014259.1:856828-859440	46.2684	4.83993	-3.25697	0.0057	down	hypothetical protein
NZ_CP014259.1:859557-861153	155.713	28.4299	-2.45341	0.0063	down	peptide ABC transporter
NZ_CP014259.1:82479-82869	140.699	2995.08	4.41191	0.00065	up	hypothetical protein
NZ_CP014259.1:861196-863879	64.9475	8.43887	-2.94415	0.03885	down	hypothetical protein
NZ_CP014259.1:861196-863879	64.2302	10.2686	-2.64501	0.0185	down	hydantoinase subunit beta
NZ_CP014259.1:863896-865543	50.9718	6.53911	-2.96253	0.0004	down	peptide ABC transporter
NZ_CP014259.1:865547-867457	61.4299	10.2078	-2.58927	0.02	down	cytochrome c550
NZ_CP014259.1:865547-867457	60.2125	10.1245	-2.57221	0.0248	down	peptide ABC transporter
NZ_CP014259.1:83020-84496	46.1752	537.542	3.54119	0.00435	up	NAD-dependent succinate-semialdehyde dehydrogenase

NZ_CP014259.1:891356-894656	596.372	58.0245	-3.36148	0.0171	down	ABC transporter substrate-binding protein
NZ_CP014259.1:88619-89480	32.982	111.979	1.76348	0.02705	up	oxidoreductase
NZ_CP014259.1:917783-918080	28.2778	157.313	2.4759	0.00255	up	photosystem reaction center subunit H
NZ_CP014259.1:89521-90544	30.3863	135.769	2.15966	0.0068	up	hypothetical protein
NZ_CP014259.1:932649-933675	9.73688	57.7242	2.56765	0.0024	up	iron ABC transporter substrate-binding protein
NZ_CP014259.1:933789-934788	9.74356	55.3669	2.5065	0.0033	up	iron ABC transporter substrate-binding protein
NZ_CP014259.1:938626-939853	16.2398	167.694	3.36822	0.00015	up	MFS transporter
NZ_CP014259.1:950737-951364	361.016	13.7782	-4.71161	5.00E-05	down	lysophospholipase
NZ_CP014259.1:951394-952729	1909.67	29.1909	-6.03166	0.00015	down	2%2C2-dialkylglycine decarboxylase
NZ_CP014259.1:93142-94147	15.97	66.621	2.06061	0.0115	up	peptide ABC transporter ATP-binding protein
NZ_CP014259.1:1006342-1008994	4.25166	12.3814	1.54207	0.0443	up	ATP-dependent DNA ligase
NZ_CP014259.1:1013445-1014222	3.94576	18.4668	2.22656	0.0113	up	exodeoxyribonuclease III
NZ_CP014259.1:1022004-1022988	21.4211	7.85184	-1.44793	0.04605	down	nitrate ABC transporter substrate-binding protein
